# Single-cell sequencing suggests a conserved function of Hedgehog-signalling in spider eye development

**DOI:** 10.1186/s13227-024-00230-6

**Published:** 2024-09-26

**Authors:** Brenda I. Medina‑Jiménez, Graham E. Budd, Matthias Pechmann, Nico Posnien, Ralf Janssen

**Affiliations:** 1 Department of Earth Sciences, Palaeobiology, Uppsala University, Villavägen 16, 75236 Uppsala, Sweden; 2 Institute for Zoology, Department of Developmental Biology, University of Cologne, Biocenter, Zuelpicher Str. 47B, 50674 Cologne, Germany; 3 Department of Developmental Biology, Göttingen Center for Molecular Biosciences (GZMB), University of Göttingen, Justus-Von-Liebig-Weg 11, 37077 Göttingen, Germany; 4 Department of Zoology, Stockholm University, 10691 Stockholm, Sweden

**Keywords:** Arthropod evolution, Arthropod head development, Eye development, Visual system development

## Abstract

**Background:**

Spiders evolved different types of eyes, a pair of primary eyes that are usually forward pointing, and three pairs of secondary eyes that are typically situated more posterior and lateral on the spider’s head. The best understanding of arthropod eye development comes from the vinegar fly *Drosophila melanogaster*, the main arthropod model organism, that also evolved different types of eyes, the larval eyes and the ocelli and compound eyes of the imago. The gene regulatory networks that underlie eye development in this species are well investigated revealing a conserved core network, but also show several differences between the different types of eyes. Recent candidate gene approaches identified a number of conserved genes in arthropod eye development, but also revealed crucial differences including the apparent lack of some key factors in some groups of arthropods, including spiders.

**Results:**

Here, we re-analysed our published scRNA sequencing data and found potential key regulators of spider eye development that were previously overlooked. Unlike earlier research on this topic, our new data suggest that Hedgehog (Hh)-signalling is involved in eye development in the spider *Parasteatoda tepidariorum*. By investigating embryonic gene expression in representatives of all main groups of spiders, we demonstrate that this involvement is conserved in spiders. Additionally, we identified genes that are expressed in the developing eyes of spiders, but that have not been studied in this context before.

**Conclusion:**

Our data show that single-cell sequencing represents a powerful method to gain deeper insight into gene regulatory networks that underlie the development of lineage-specific organs such as the derived set of eyes in spiders. Overall, we gained deeper insight into spider eye development, as well as the evolution of arthropod visual system formation.

**Supplementary Information:**

The online version contains supplementary material available at 10.1186/s13227-024-00230-6.

## Background

**Fig. 1:**
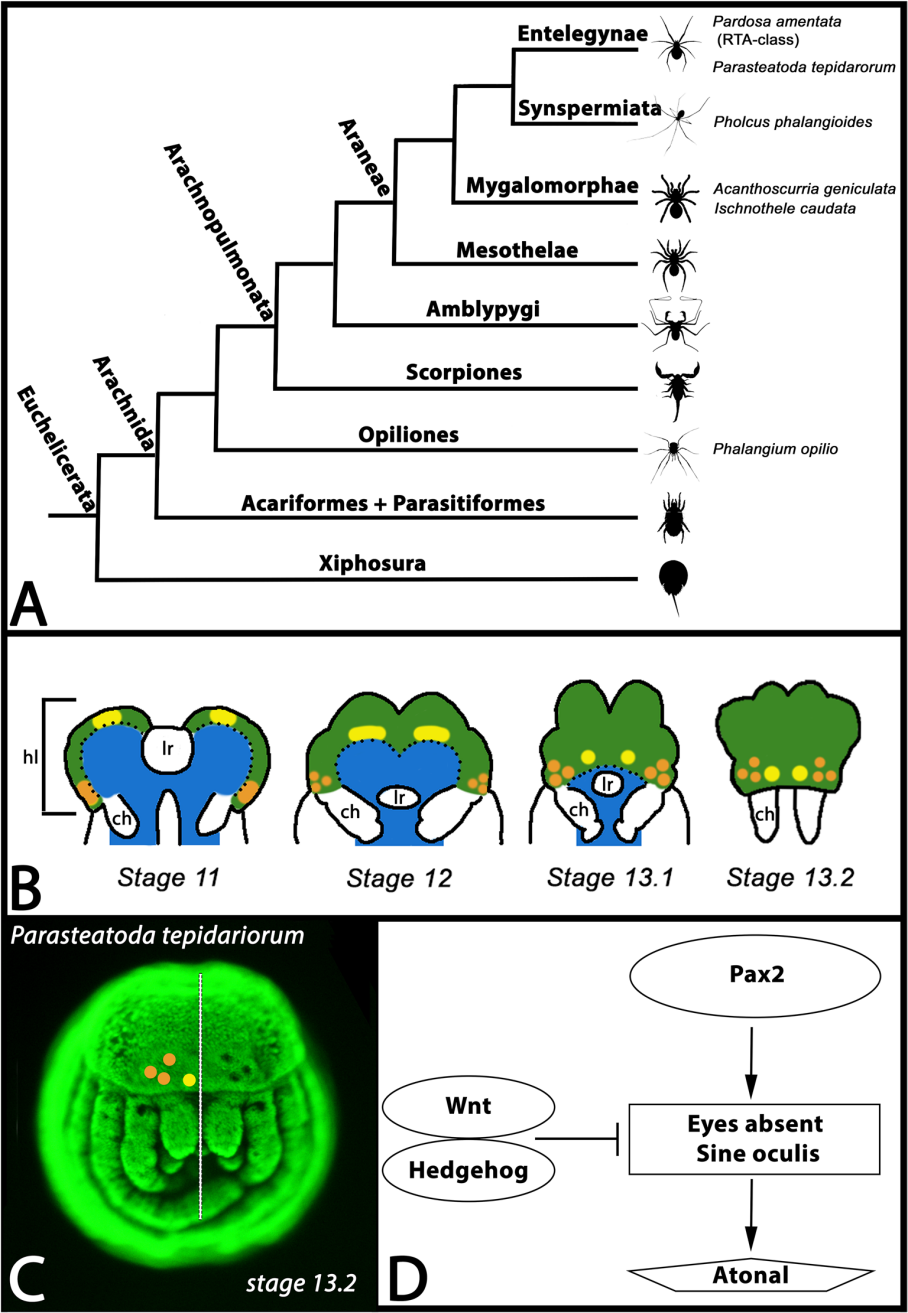
Cladogram of chelicerate phylogeny, spider eye development, and the hypothesized spider eye gene regulatory network (GRN). **A** Our study includes species representing three of the four main groups of Araneae, Entelegynae (*Parasteatoda tepidariorum*, *Pardosa amentata*), Synspermiata (*Pholcus phalangioides*), and Mygalomorphae (*Acanthoscurria geniculata* and *Ischnothele caudata*). Note that *Pardosa* represents the RTA-class of entelegyne spiders. The animal sketches come from https://www.phylopic.org. **B** Schematic overview of spider eye development. The primary eyes (yellow) develop from the non-neurogenic ectoderm (green) at the anterior rim of the head lobes. The secondary eyes (orange) develop from the non-neurogenic ectoderm (green) anterior and lateral to the base of the chelicerae (ch). The neurogenic ectoderm is depicted (blue). Additional abbreviations: hl, head lobe; lr, labrum. **C** Sybr-green stained stage 13.2 embryo of the spider *Parasteatoda tepidariorum*. On the left half of the embryo (dashed line marks the symmetry axis), the secondary eyes (orange) and the primary eyes (yellow) are indicated. Note the grooves where the secondary eyes form (visible on the right half of the embryo). **D** Suggested eye developmental GRN in spiders. Based on comparative gene expression data from Baudouin-Gonzalez et al. [6] and Janeschik et al. [34]

The ability to process information provided by light is pervasive in animals as it represents an evolutionary advantage [52]. Starting with simple light-sensitive cells or organs, complex eyes evolved already more than half a billion years ago in the Cambrian (e.g. [3, 11, 24, 53, 87]) and may have contributed to the “Cambrian explosion” (e.g. [74]). Among arthropods, insects and spiders (Araneae) (Fig. 1A) have evolved the most complex eyes (e.g. [51], Morehouse 2020). Most spiders possess eight eyes, except for some groups that possess only six [51] or less, often due to a loss of some of the secondary eyes (e.g. [33]). The four pairs of eyes of (most) spiders are named after their position on the spider’s head, i.e. the anterior median (AM) eyes that represent the principal/primary eyes of spiders. In contrast, the anterior lateral (AL), the posterior lateral (PL), and the posterior median (PM) eyes represent the secondary eyes of spiders (Fig. 1B). There is accumulating evidence that the principal eyes of spiders are homologous with the ocelli of insects, and that the secondary eyes of spiders are homologous with the compound eyes of insects (reviewed in [63], Morehouse 2020). The three secondary pairs of eyes indeed likely evolved from compound eyes present in their aquatic ancestors albeit reducing the number of facets from about a couple of thousands to only three [63, 67].

Principal and secondary eyes of spiders both originate from the non-neurogenic ectoderm of the head lobes, but from two different regions: the principal eyes originate from the anterior edge of the head lobes, while the secondary eyes form at the lateral edge of the posterior head lobe close to the base of the chelicerae (e.g. [6, 77, 79]) (Fig. 1B). Later during development, the non-neurogenic ectoderm overgrows the neurogenic ectoderm translocating especially the principal eyes into a more anterior-facing position (Fig. 1B, C). The final position of the eyes, however, can vary in different groups of spiders (reviewed in [7, 63, 64, 86]. One of the main differences between principal and secondary eyes of spiders is that the retinas of the principal eyes are everted, while the retinas of the secondary eyes are inverted, suggesting different evolutionary origin of these two types of eyes [30]. The gene regulatory network underlying arthropod eye development appears to be widely conserved (e.g. [89]) and is thus far best studied in the vinegar fly *Drosophila melanogaster* (reviewed in e.g. [12, 18, 50, 92]). Interestingly, although a core gene regulatory network (GRN) appears to contribute to the development of all types of light-sensitive organs (eyes) in the fly, i.e. ocelli, compound eyes and Bolwig’s organs, there are still differences in gene regulation and interaction in the different types of eyes (reviewed in e.g. [18]). In a nutshell, *Pax6* genes determine the optic fields in which the compound eyes and the ocelli develop, and they activate the retinal determination genes (RDGs) such as *eyes absent* (*eya*), and *sine oculis* (*so/six1*) ([18, 90]. Wnt-signalling inhibits eye development and Wnt gene expression thus restricts the expansion of the optic field (e.g. [54]). Downstream of the RDGs, and additionally activated by Hedgehog (Hh)-signalling, acts the proneural gene *atonal* (*ato*) in the developing eyes of *Drosophila* [46] that finally activates the expression of the photoreceptor specific gene *glass* (*gl*) [65].

In the common house spider *Parasteatoda tepidariorum*, *Pax6* appears to be replaced by another Pax gene *Pax2*, at least in the secondary eyes [34], and Wnt-signalling likely represses the development of eyes [6]. Like in *Drosophila*, *eya* and *so/six1* are expressed in all spider eyes ([20, 77, 79]), and also the RDG downstream factor *ato* is expressed in all eyes of *Parasteatoda* [6] (Fig. 1D). Data on *gl* expression in spiders are lacking, as well as data on Hh-signalling that would suggest a conserved function during the activation of retinal target genes. Instead, in *Parasteatoda*, gene expression studies suggest that not only Wnt-signalling but also Hh-signalling appear to restrict the eye fields and consequently that the eyes develop in regions of the head where neither Wnt- nor Hh-signalling occurs [6] (Fig. 1D).

We previously performed single-cell sequencing (SCS) in late-stage embryos of *Parasteatoda* revealing several cell clusters with a neurogenic fingerprint, including clusters representing the developing peripheral and sensory nervous system [60]. In the current study, we provide an improved analysis of this previous data using newer versions of analytical software and more restricted criteria for the definition of marker genes. The new analysis revealed a previously undetected cell cluster with an eye-specific fingerprint represented by numerous known *Parasteatoda* eye-marker genes (cf. e.g. [7, 79]). Interestingly, we found that the most specific genetic marker of this cluster is represented by a previously unstudied paralog of *hh*. In this paper we investigate the top markers of this cluster, including the previously unstudied *hh* paralog, and show that they are expressed in the developing eyes of *Parasteatoda*. For *hh* genes, we expanded our study to representatives of all main groups of spiders (Fig. 1A) demonstrating that Hh-signalling is involved in eye development in spiders as a whole (taking mRNA expression of the sole ligand of this pathway, *hh* (or its paralog *hh2*), as evidence for pathway activity [47]. Beyond that important finding, our data also identify additional genes that are expressed during spider eye development but that have not been studied in this context yet. Our data thus contribute to a deeper insight into eye development in spiders.

## Methods

### Research animals

The embryos of *Parasteatoda* come from our own culture in Uppsala. Embryos of *Acanthoscurria* and *Ischnothele* come from the culture of Matthias Pechmann (Cologne University). Fertilized females of *Pholcus* were collected in the basement of a house, 47559 Kranenburg, Germany, and now serve as the founders of a laboratory colony in Uppsala. Egg sacks were collected from females of *Pardosa* at the KFUM Uppsala Survival trainings facility, 75260 Uppsala, Sweden, during July and August 2021. Females of *Pardosa* were released back into the wild after carefully collecting their egg sacks. Embryos of the harvestman *Phalangium opilio* were obtained and treated as described in Janssen et al. [41].

### Single-cell sequencing

Embryonic tissue dissociation, cell capture, cDNA library preparation, and single-cell RNA sequencing were performed at the Department of Developmental Biology and Gene Core facilities of the European Molecular Biology Laboratory (EMBL), Heidelberg, Germany. These processes as well as quality control of sequencing reads, and reference genome pre-processing are described in Medina-Jiménez et al. [60]. The raw sequencing reads used in the present study are the same as presented in Medina-Jiménez et al. [60]. We here present a de novo gene expression matrix generated after mapping the sequencing reads to the reference genome Ptep_3.0 (same version of the genome as in Medina-Jiménez et al. [60] using CellRanger version 7.0.1. instead of the previously used CellRanger version 6.0.2 (Supplementary File 1). Unlike the previous study, we did not apply any UMI number restrictions in the de novo analysis. The resulting gene expression matrix was loaded into Seurat ver. 4.3.0, an R package for analysis of single-cell RNA sequencing [25, 78]. Only cells with at least 250 expressed genes (min.features = 250), with no more than 5% of these genes being mitochondrial, and genes that are expressed in at least three cells (min.cells = 3) were kept for downstream analysis. Filtering of low quality, and non-informative cell barcodes was done by a back-and-forth strategy, in which cells were processed up to the clustering and marker selection steps, then clusters with markers that did not pass the pre-established threshold were removed, and the cells were processed again. Data normalization, variance stabilization, linear and non-linear dimensional reduction and clustering was done as in Medina-Jiménez et al. [60]. The Seurat function ‘FindAllMarkers’ was used to identify the differentially expressed genes (DEGs) to be used for cluster identification (Supplementary File 2). Parameters included selecting only the DEGs that are upregulated (only.pos = TRUE), and those that are expressed in at least 10% of the cells in a cluster (min.pct = 0.1). In addition, a return threshold of 1e^−10^ was established to avoid selecting markers that have a high adjusted p-value. The final subset is available in *.rds format (Supplementary File 3).

### Gene orthology and gene ontology (GO) analysis

In order to identify *Drosophila melanogaster* orthologs of the cluster markers, we conducted reciprocal best hits (RBH) using the software MMSeqs2 [85]. For the gene ontology analysis of these orthologs, we applied the bioinformatics package clusterProfiler [97] using the function ‘enrichGO’ library and the *Drosophila melanogaster* database (or.Dm.eg.db) and focusing only on the analysis of GO: Biological Processes.

### Phylogenetic analyses

*Hedgehog* (*hh*) genes were identified in the genome of the true spider *Parasteatoda tepidariorum* [80] and the transcriptomes of the true spiders *Cupiennius salei* [77], *Pardosa amentata* [26] and *Pholcus phalangioides* [39], the mygalomorph spiders *Acanthoscurria geniculata* [73] and *Ischnothele caudata* (TRINITY assembled transcriptome, embryonic stages 5–14 and the postembryonic stage), and the harvestman *Phalangium opilio* [81] performing reciprocal BLAST searches (tBLASTn) using the Hedgehog protein sequence from the vinegar fly *Drosophila melanogaster* as query. A phylogenetic analysis was performed using MrBayes [31] in the same way as previously described in Panara et al. [70]. Amino acid sequences of the complete coding regions of *hh* genes (as far as available) were aligned using T-Coffee with default parameters in MacVector v12.6.0, followed by manual editing (Supplementary Files 4–6). All phylogenetic analyses conducted in this paper were performed using a fixed WAG amino acid substitution model with gamma-distributed rate variation across sites (with four rate categories), unconstrained exponential prior probability distribution on branch lengths, and exponential prior for the gamma shape parameters for among-site rate variation was applied. Gene topology of the Hh-tree was analysed applying 1 million cycles for the Metropolis-Coupled Markov Chain Monte Carlo (MCMCMC) analysis (four chains; chain-heating temperature of 0.2). Markov chains were sampled every 200 cycles and default settings of 25% of samples were applied as burn-in. Clade support was calculated with posterior probabilities in MrBayes. The resulting tree was midpoint rooted.

We also performed a phylogenetic analysis with the identified CD36-family gene of *Parasteatoda* that we named *CD36.1*, additional closely related CD36-family genes of *Parasteatoda*, CD36-family genes from the zebrafish *Danio rerio* and the house mouse *Mus musculus* [98], and all previously identified *Drosophila melanogaster* CD36-family genes [66], using the same parameters as applied for the Hh-analysis (see above) but with 0.5 million cycles for the MCMCMC analysis (Supplementary Files 7 and 8). Gene identifiers are shown in the tree (Supplementary File 9).

The phylogenetic analysis of spider T-box genes including *optomotor-blind* (*omb*) genes has been published in Janssen and Budd [45].

### In situ hybridization and data documentation

Fragments of all investigated genes were amplified by means of PCR using gene specific primers (Supplementary File 6). In all cases, a T7 RNA-polymerase promotor site (TAATACGACTCACTATAG) was added to the reverse primer to allow a direct use of the PCR amplicon as template for subsequent anti-sense RNA-probe synthesis [13]. Spider embryos and harvestman embryos were fixed for in situ hybridization as described in Prpic et al. [76] (for spiders) and Janssen et al. [41] (for the harvestmen) at room temperature on a moderately moving tumbling table for 16 to 24 h. All in situ hybridizations were performed as described in Janssen et al. [40]. All embryos were incubated in 1:10,000 Sybr-green in phosphate buffered saline pH 7.4 with 0.1% Tween-20 (PBST) for 30 min. Unincorporated Sybr-green was removed by washing with PBST (washing 3 times for 10 min on a tumbling table). Pictures of stained embryos were taken under a MZ-FLIII Leica dissection microscope equipped with a Leica DC490 digital camera and an external UV-light source. Linear adjustments were made using the image-processing software Adobe Photoshop 2022.

## Results

### Improved analysis of single-cell sequencing (SCS) data reveals new cell clusters and further specifies previously discovered cell clusters

**Fig. 2:**
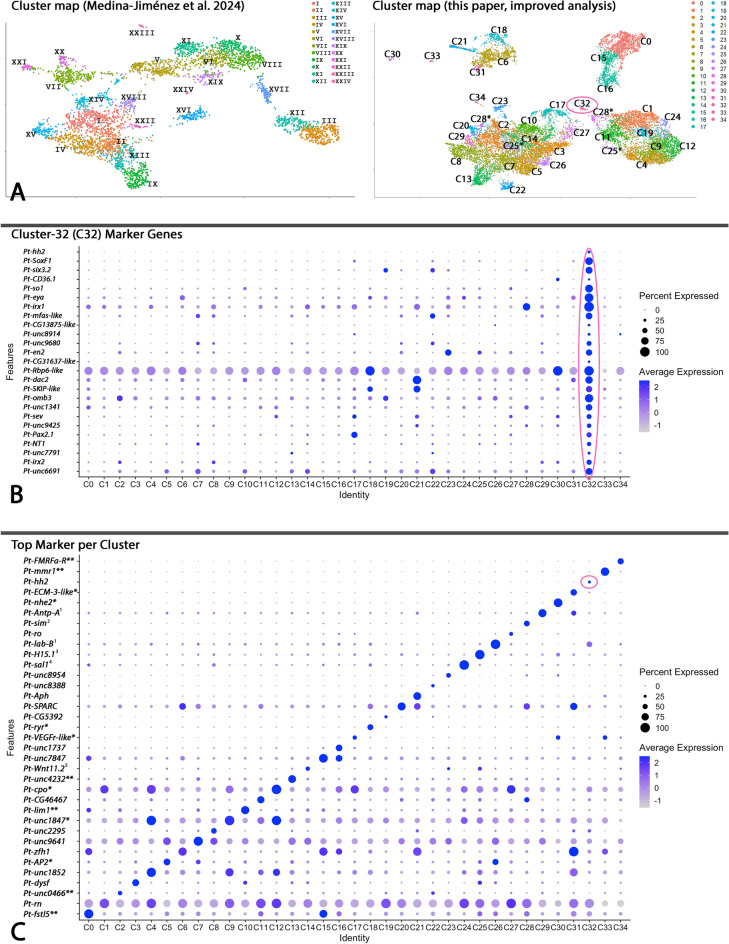
**A** Cluster map comparison Medina‑Jiménez et al. [60] *vs* this paper, **B** dot plots of the Cluster-32 (C32) marker genes, **C** the top marker per cluster. The cluster maps in **A** represent two-dimensional projections of three-dimensional systems. Clusters marked with asterisks represent clusters that appear to be separated, but this is an optical artefact of the 2D/3D effect. The eye-specific cluster C32 is encircled (**A**), and so are the markers that are correlated with this cluster (**B** and **C**). Note that the clusters VIII and XIX were incorrectly labelled in the UMAP provided by Medina‑Jiménez et al. [60]; we corrected this mistake in panel **A**. Available embryonic gene expression data of the best markers per cluster are indicated: * Medina et al. [60]; ** Supplementary File 10; ^1^ Schwager et al. [80],^2^ Linne et al. [57]; ^3^ Janssen et al. [36],^4^ Janssen et al. [45]; ^5^ Janssen et al. [41]

Previously, we performed SCS on complete *Parasteatoda* embryos representing the developmental stages 10–12 (after [61]) in which we identified several cell clusters assigned to the developing central and peripheral nervous system [60]. Based on this pioneering study, we improved our data analysis that we publish in the current paper. This led to the discovery of new cell clusters (Fig. 2; Table 1) including new marker genes (Supplementary File 2), and the improvement of the specific genetic fingerprints of previously recovered cell clusters (cf. [60]). We based our cluster-predictions on the embryonic gene expression patterns of previously published marker genes (Table 1), newly provided expression data (Supplementary File 10), and a GO analysis (Supplementary Files 11–13). The new GO analysis provides information on the putative biological function of the cells in a given cell cluster, although this information has to be handled with care given the possibility of research biases such as above average interest in certain biological functions. This can lead to an above average of available comparative information on such a topic (and associated genes). And this can possibly lead to incorrect cluster characterization. A potential example is given by cluster C25 that according to our GO analysis would likely represent cells involved in cardiac development, although available gene expression data rather suggest another location of these cells (cf. Table 1). Whole mount in situ hybridization data that provide spatial expression data within the developing spider embryos are thus required to verify (or falsify) literature and GO analysis predictions.

**Table 1:** Cluster identification and comparison

Clusters		Cluster identity		Markers
CellRanger 7 This paper	CellRanger 6 [60]	[60]	Newly identified clusters in this paper and new specifications	WISH presented in [60]*, Supplementary File 10**, and other publications
C0	III	CNS-1 (axon development and guidance-1)		*fstl5*(LOC107441591)* *sax3*(LOC107455609)* *pika*(LOC107439395)* *Ncad2*(LOC107451854)* *Ncad1*(LOC107454545)*
C1	XI	CNS-6 (optical system development-1)		*cpo*(LOC107453124)* *delta-2*(LOC107438011)* *N*(LOC107450518)** *dac1*(LOC107453438)^a^ *SoxN*(LOC107457313)^b^
**C2**	—	—	EctoAppen-8: proximal of appendages, opisthosoma (?)	*unc5.2*(LOC107437991)^d^ *opa*(LOC107437305)** *unc0466*(LOC122270466)** *Ubx*(LOC107457033)^e^ *abdA*(LOC107450666)^e^
C3	II + XV	EctoAppen-2 + 5: joints		*disco*(LOC107447159)* *odd*(LOC107444970)^f^ *trh1*(LOC107455153)^g^ *al*(LOC107448374)^g^
C4	VIII	CNS-4		*cpo*(LOC107453124)* *Dl*(LOC107438011)* *Nkx6.2*(LOC107450777)* *unc1847*(LOC107451847)*
C5	XV	EctoAppen-5: prosomal appendages		*AP2.2*(LOC107443623)* *Dll*(LOC107450100)* *Dfd*(LOC107444120)^e^ *trh1*(LOC110283103)^g^ *cll2*(LOC107451627)^g^
C6	VII	Mesoderm-EMT (1)		*PS2*(LOC107444850)* *papilin*(LOC107447945)* *fibrinogen-like*(LOC107449293)*
C7	IV + XIII	EctoAppen-3 + 4: possibly joint-related		*np*(LOC107439948)* *endoA*(LOC107442511)* *elovl7.2*(LOC107441276)*
C8	I	Dorsal tissue/EctoAppen-1		*trh1*(LOC110283103)^g^ *elovl7*(LOC107446178)* *hex1*(LOC107442242)*
C9	X	CNS-5		*unc1847*(LOC107451847)* *Dl*(LOC107438011)*
**C10**	—	—	EctoAppen-9: proximal of appendages	*lim2*(LOC107449891)** *lim1*(LOC107442352)** *exd1*(LOC107449226)^h^ *unc5.2*(LOC107437991)^d^
C11	V	CNS-2 (axon development and guidance-2)		*zfp395-like*(LOC122271089)* *nog*(LOC107444265)* *magu*(LOC107453771)* *net1*(LOC107450632)^d^
C12	VI	CNS-3		*cpo*(LOC107453124)* *unc1847*(LOC107451847)* *CD109*(LOC107436484)* *Dl*(LOC107438011)*
C13	IX	Opisthosomal appendages: spinnerets (cuticle-development and moulting related)		*unc4232*(LOC107454232)** *unc0558*(LOC107440558)* *unc2247*(LOC107452247)*
**C14**	—	—	EctoAppen-7(1): Ventral side of appendages	*Wnt11.2*(LOC107446521)^i^ *lbx*(LOC107442844)^j^ *Wnt1*(LOC107438386)^i^ *Wnt6*(LOC107438387)^i^
C15	XII	CNS-7 (axon development and guidance-3)		*unc3142*(LOC107443142)* *dpr6*(LOC107436606)* *Ncad2*(LOC107451854)* *scrtl*(LOC107456893)*
C16	XVII	CNS-8 (axon development and guidance-4)		*insc*(LOC122271737)* *pros*(LOC107448306)^k^ *brat*(LOC107438139)* *nerfin*(LOC107446363)*
C17	XVI	PNS-1 (optical system development-2)		*VEGF*(LOC107454058)* *ss*(LOC107454134)* *ss2*(LOC107457395)* *sev*(LOC107441450)*
C18	XX	Heart		*ryr*(LOC107450496)* *rapk2*(LOC107453369)* *lethal2*(LOC107453784)* *Mef2*(LOC107445920)^a^
**C19**	—	—	Brain-1: optical system development-3	*Dmrt99B*(LOC107454113)^m^ *six3.2*(LOC107436457)^n^ *tll*(LOC107444665)^o^ *scro*(LOC107436846)**
C20	XVIII	EctoAppen-6: EMT (1) (“tube” development-1)		*papilin-like*(LOC122270011)* *PS1*(LOC107454271)* *nord*(LOC107451942)*
C21	XXI	Midgut/yolk/hematopoiesis/immune response		*unc8180* (107448180)* *NaK-t-ATPase*(LOC107456567)* *aqp7l*(LOC107457171)*
C22	XXII	Stomodaeum-1		*vsx*(LOC122268388)* *unc5848*(LOC107445848)* *unc3843*(LOC107453843)* *unc3221*(LOC107453221)**
**C23**	—	—	Stomodaeum-2	*scro*(LOC107436846)** *Dmrt93B*(LOC107454114)^m^ *FoxA-2*(LOC107452746)^15^ *unc7000*(LOC107447000)**
**C24**	—	—	Brain-2 optical system development-4	*sal1*(LOC107450899)^q^ *Pax6.2*(LOC107441893)^n^ *gsc*(LOC107439785)** *otd2*(LOC107457564)^n^
**C25**	—	—	EctoAppen-7(2): ventral side of appendages	*H15.1*(LOC107436847)^c^ *Wnt11.2*(LOC107446521)^g^ *Wnt6*(LOC107438387)^g^ *Wnt1*(LOC107438386)^g^
C26	XV	EctoAppen-5 (2): pedipalps (male genitalia?)		*lab-B*(LOC107440711)^e^
**C27**	—	—	PNS-2 (optical system development-5)	*cpo*(LOC107453124)* *SoxF2*(LOC107454275)^q^
C28	XIV	Midline/ventral sulcus (axon development and guidance-5)		*sim*(LOC107439751)^r^ *net1*(LOC107455212)^d^ *sideVIII*(LOC107456176)*
**C29**	—	—	Book lungs / tracheae	*Antp-A*(LOC107447498)^e^ *Antp-B*(LOC107450669)^e^ *sal2*(LOC107447905)^p^
C30	XXIII	Midgut/yolk		*nhe2*(LOC107452408)* *mrp1*(LOC107440407)* *PiT1-like*(LOC107453365)*
**C31**	VII	Possibly mesoderm or EMT (2) (“tube” development-2)		no available WISH data
C32	XIX	CNS-9: eyes		THIS PAPER
**C33**	—	—	Immune system	*mmr1*(LOC107452426)** *mmr2*(LOC107446703)**
C34	XXIV	Limb innervation		*FMRFa-R*(LOC107448206)** *nrf6-l*(LOC107448146)* *elovl4-like*(LOC122269995)*

^a^Turetzek et al. [91]

^b^Bonatto Paese et al. [8]

^c^Janssen et al. [36]

^d^Janssen and Budd [44]

^e^Schwager et al. [80]

^f^Heingård et al. [28]

^g^Zhang [99]

^h^Schomburg et al. (2020)

^i^Janssen et al. [41]

^j^Aase-Remedios et al. (2023)

^k^Weller and Tautz [96]

^l^Leite et al. [56]

^m^Panara et al. (2019)

^n^Schomburg et al. [79]

^o^Janssen et al. [43]

^p^Medina‑Jiménez et al. [59]

^q^Baudouin-Gonzalez et al. [5]

^r^Linne et al. [57]

^**^Supplementary File 10

In the new analysis, we identified the novel clusters C2 and C10 (both putatively representing the proximal ectoderm of developing appendages), C14 and C25 (both putatively representing the ventral ectoderm of the appendages), C19 (putatively representing a subset of the developing brain), C23 (putatively, a second cluster representing the developing stomodaeal area), C24 (putatively a second brain-specific cluster), C26 (putatively representing tissue of the pedipalps and/or the pedipalpal segment), C27 (putatively a second cluster representing the peripheral nervous system), C29 (putatively representing the book lungs and the tracheae), and C33 (putatively representing the immune system) (Table 1).

With the aid of the GO analysis, we also managed to further specify previously identified and new cell clusters. For example, clusters C0, C11, C15, C16, and C28 that were previously simply described as associated with the CNS and the midline/ventral sulcus can now be more accurately defined as also be involved in axon-guidance (Table 1; Supplementary Files 11–13). Clusters C1, C17, C19, C24, C27 appear to be generally involved in visual system development, although their marker genes are not necessarily expressed in the developing eyes themselves (examples provided in Supplementary File 10, see panels O, P, S).

We identified *inter alia* a small cell cluster (C32) represented by 39 cells and 25 differentially expressed marker genes (Fig. 2A; Supplementary Files 2 and 6). In our previous study, this cell cluster was named Cluster-XIX and described as “*sensory nervous system of the head*”-specific cell cluster (CNS-9) (Table 1); this cluster was represented by 79 cells and was defined by 42 marker genes [60]. Many of the marker genes of C32 have previously been studied in the context of spider eye development including *six3.2*, *so1*, *eya*, *dac2*, and *Pax2.1* (e.g. [79]), suggesting that this cluster indeed specifically represents the developing eyes, rather than anterior sensory organs in general. Our present GO-analysis further substantiated this assumption (Supplementary File 11).We provide feature-plots of all 25 marker genes of cluster C32 (Supplementary File 14).

The most specific marker gene of C32 is *hedgehog* 2 (*hh2*), a paralog of the previously studied segment-polarity gene *hedgehog* (*hh*) (hereafter referred to as *hh1*). Intriguingly, this gene has not been addressed by earlier studies.

### Paralogs of *hedgehog*

**Fig. 3:**
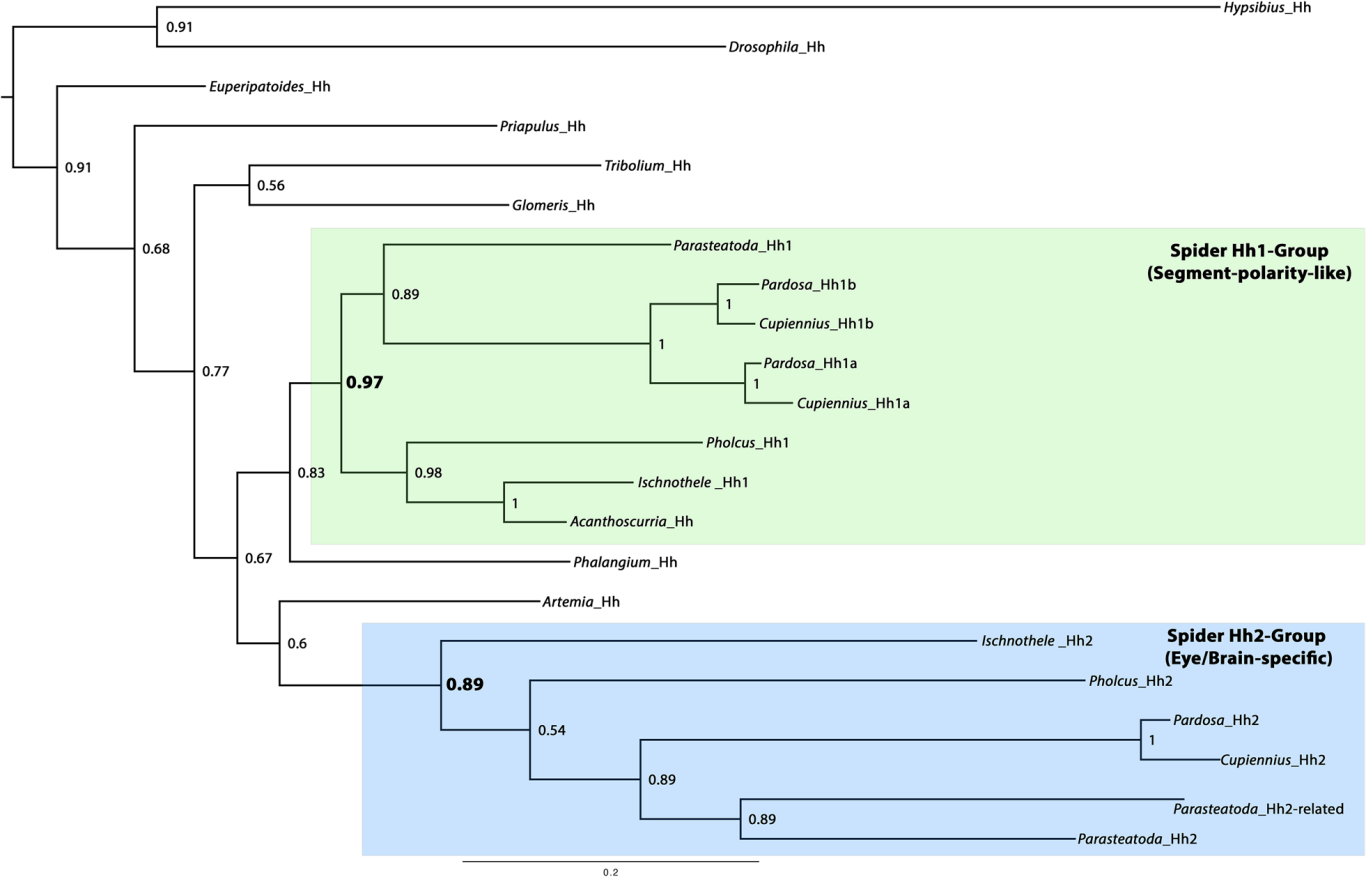
Phylogenetic tree of spider *hedgehog* (*hh*) genes. Bayesian analysis using MrBayes applying 1 million cycles for the Metropolis-Coupled Markov Chain Monte Carlo (MCMCMC). The tree is midpoint rooted. Node labels represent posterior possibilities. The scale bar represents 0.2 amino acid substitutions per site. Note that the two groups of spider *hh* genes (Group-1, Hh1 and Group-2, Hh2) separate with almost total support. Species information: *Acanthoscurria geniculata*; *Artemia franciscana* (Branchiopoda); *Cupiennius salei*; *Drosophila melanogaster* (Insecta); *Euperipatoides kanangrensis* (Onychophora); *Hypsibius exemplaris* (Tardigrada); *Glomeris marginata* (Myriapoda); *Ischnothele caudata*; *Pardosa amentata*; *Parasteatoda tepidariorum*; *Phalangium opilio* (Opiliones); *Priapulus caudatus* (Priapulida); *Tribolium castaneum* (Insecta)

We identified three copies of *hh* in the transcriptomes of the entelegyne spiders *Cupiennius* and *Pardosa*, and the genome of the entelegyne spider *Parasteatoda*, two copies in the transcriptome of the synspermiatan spider *Pholcus*, and two copies in the transcriptome of the mygalomorph spider *Ischnothele* (Figs. 1A and 3). In another Mygalomorpha, *Acanthoscurria*, however, we only identified one copy of *hh* (Fig. 3). The latter likely represents a shortcoming of the sequenced embryonic transcriptome of *Acanthoscurria* rather than a gene loss, given that two copies exist in *Ischnothele*; a loss of *hh2* in *Acanthoscurria* can, however, not be excluded.

Our phylogenetic analysis indicates that one spider *hh* paralog (Fig. 3, green background, referred to as the “*hh1*-group”) has been duplicated once more in RTA-class spiders represented by *Cupiennius* and *Pardosa*, but not so in the other groups of spiders (Figs. 1A and 3).

The second paralog of *hh* (Fig. 3, blue background, referred to as the “*hh2*-group”) duplicated in *Parasteatoda*, but not in the other investigated spiders. One of these copies underwent drastic changes losing the ‘Hedge’ domain and instead acquired two N-terminal EGF domains (Supplementary File 4) (see [10] for a review on *hh* and *hh*-related genes and their protein motifs).

In representatives of other groups of arthropods, an onychophoran, a tardigrade and a priapulid, we only identified one single copy of *hh*. Note that these sequences do not cluster according to animal phylogeny. The *hh1* and *hh2* groups of spiders, however, each form distinct clusters (Fig. 3). The source of this could be a whole genome duplication as suggested by some authors (e.g. [80, 82]), but in the case of *hh*, this would require more extensive analyses.

### Expression of spider *hedgehog* (*hh*) genes

Expression of one *hh* paralog (i.e. *hh1*) has been described in detail in the spider *Parasteatoda* [2, 6, 15, 48, 69, 72].

All here identified *hh1* genes in the investigated spiders are expressed in virtually the same pattern as in *Parasteatoda*, including the SPG-like expression in the form of transverse segmental stripes, expression in the appendages, dynamic expression in the SAZ, and expression in the stomodaeum and the pre-cheliceral region (Supplementary File 15). In *Pardosa*, that possesses two copies of *hh1* (*hh1a* and *hh1b*), *hh1a* is not expressed in the SAZ during the process of segment addition (Supplementary File 15, panel B). In *Acanthoscurria* there are four small dots of expression anterior to the labrum in the neuronal ectoderm at late developmental stages; this expression, however, is not associated with the developing eyes (Supplementary File 15, panel O). *hh1* is thus not expressed in the developing eyes of *Parasteatoda* [6] or any other investigated spider.

**Fig. 4:**
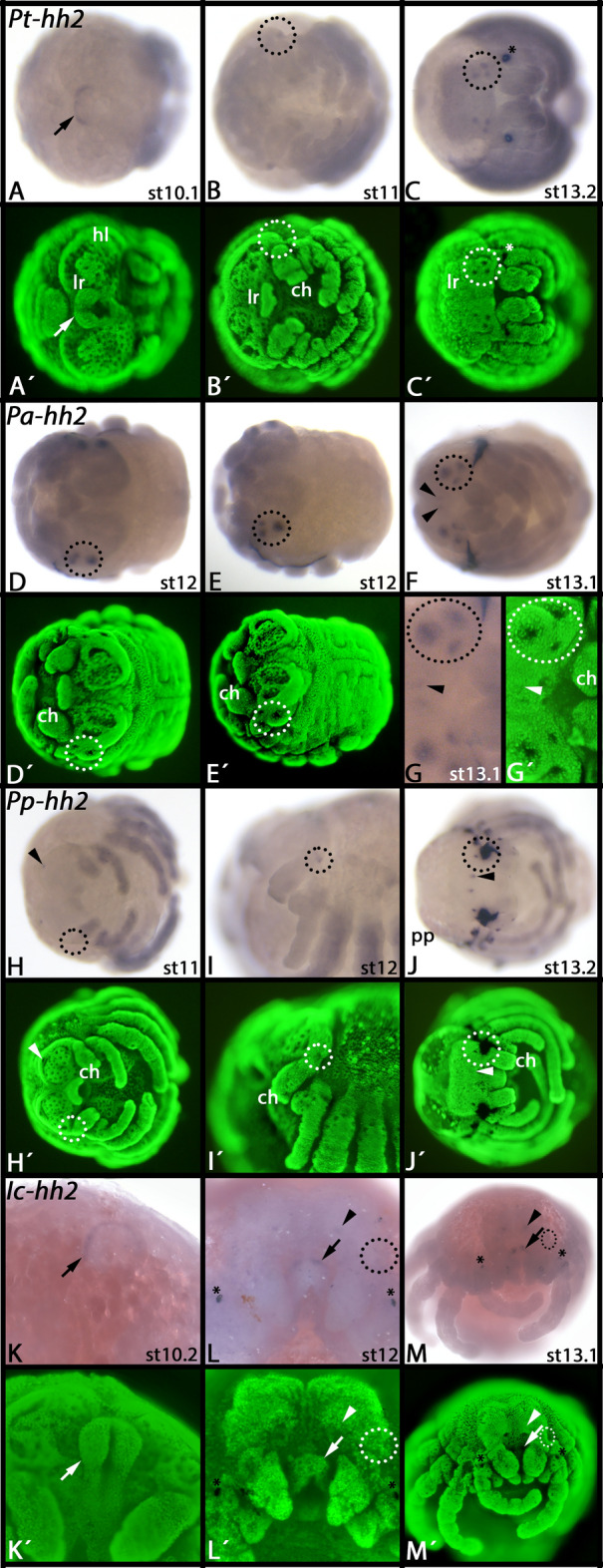
Expression of spider *hh2* genes. In all panels, anterior is to the left, except panels** K**-**M** (anterior up). The arrows in panels** A**,** K**,** L**, and** M** point to expression in the labrum. In all panels, arrowheads point to expression in the primary eyes. The secondary eye primordia are encircled (dashed circles and ellipses). Panels marked with an apostrophe (´) represent Sybr-green staining of embryos shown in corresponding bright field images. Developmental stages are indicated after Mittmann and Wolff [61]; note that developmental stages in different species of spiders have been defined by comparable morphological landmarks such as the overall shape of the embryo and the length of the appendages

In all investigated spiders, *Parasteatoda*, *Pardosa*, *Pholcus* and *Ischnothele*, expression of *hh2* genes is restricted to regions of the developing head (with the exception of the duplicated *hh2-related* gene of *Parasteatoda*). In *Parasteatoda*, *hh2* is transiently expressed in the developing labrum (Fig. 4A). At around stage 10.2 (after [61]), expression of *hh2* starts in the form of two dots laterally on either side of the head lobes in the non-neurogenic ectoderm in the place where the primordia of the secondary eyes are located (Fig. 4B). Later, this expression transforms into six distinct dots that represent the six secondary eyes (Fig. 4C). We did not detect any expression of *hh2* in the principal eyes of *Parasteatoda*.

In *Pardosa*, we detect a similar pattern in the secondary eyes (Fig. 4D–F), but we also detect faint expression of *hh2* in the principal eyes at later developmental stages (Fig. 4F, G).

In *Pholcus*, we find weak dot-like expression in the lateral non-neurogenic ectoderm that is likely associated with the developing secondary eyes (Fig. 4H–J). Moreover, we detect tiny dots of expression anterior in the non-neurogenic ectoderm where the primordia of the principal eyes are located (Fig. 4H, J). Later during development, we see strong expression in the principal and the secondary eyes (detection of the latter is, however, inhibited in two of the three secondary eyes due to the relatively early development of the cuticle that stains un-specifically and covers at this stage two of the three secondary eyes at either side of the head lobe) (Fig. 4J).

In *Ischnothele*, like in *Parasteatoda*, *hh2* is expressed in the labrum (Fig. 4K–M). At late developmental stages, *hh2* also is expressed in the primordia of the primary eyes (Fig. 4L, M) and weakly in the developing secondary eyes (Fig. 4L, M); the latter, however, is almost below detectable level at the stages for which we have embryos.

Although we did not detect expression of *hh2* in the labrum in *Pardosa* and *Pholcus*, this could simply be due to the lack of embryos representing all developmental stages, including the stage during which *hh2* is expressed in the labrum.

The duplicated and modified *hh2*-class gene found in *Parasteatoda*, referred to as *Parasteatoda-hh2-related* (*Pt-hh2-r*) is expressed in the dorsal field, and hence is likely involved in either midgut development (Supplementary File 16, panels A and B) (cf. [15]), yolk-metabolism, or the innate immune system (cf. [60]).

### Expression of the *Phalangium opilio hedgehog* (*hh*) gene

**Fig. 5:**
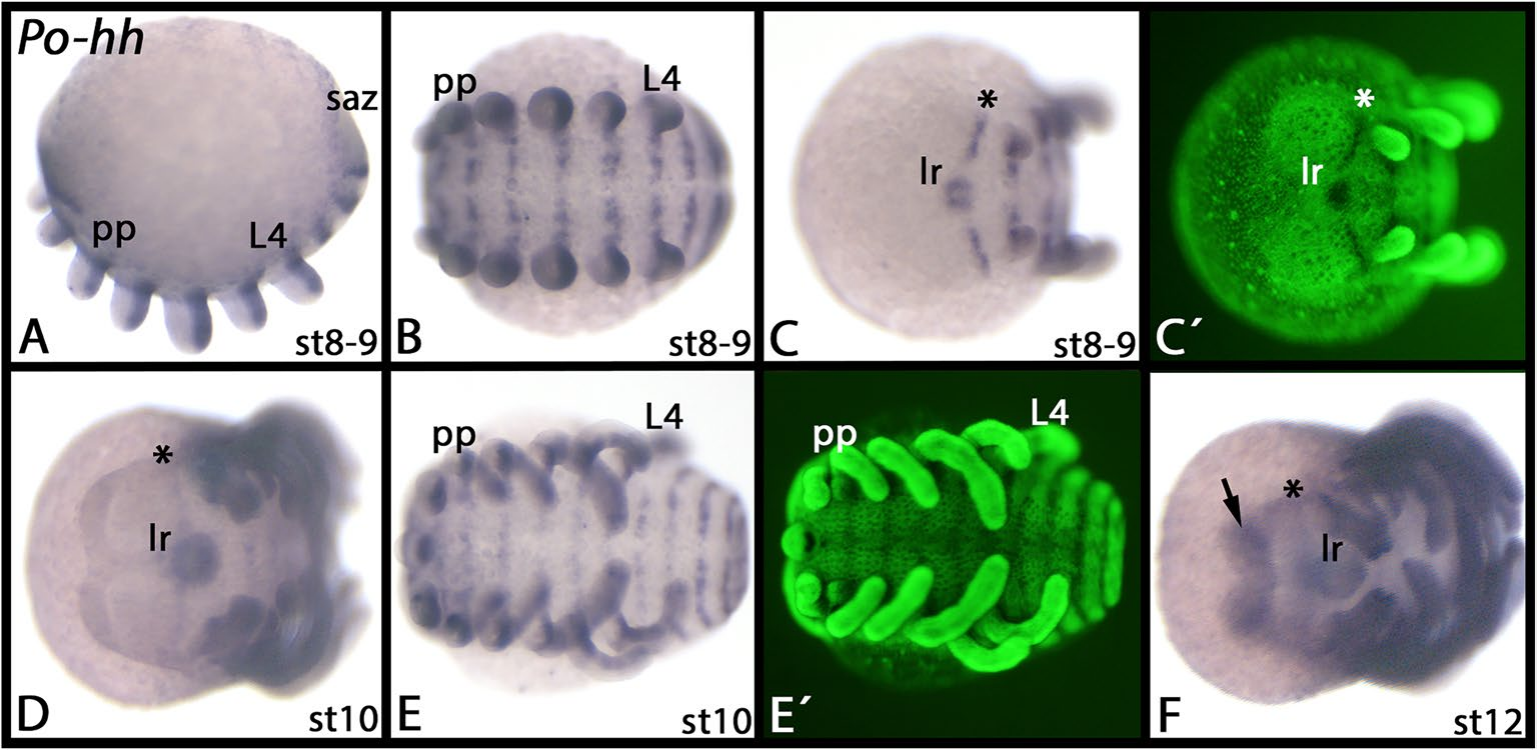
Expression of *Phalangium opilio hedgehog* (*hh*). In all panels, anterior is to the left. Panel **A** represents a lateral view (dorsal up). Panels **B** and **E** represent ventral views, and panels **C**, **D** and **F** represent views onto the head region. The asterisks in panels **C**, **D** and **F** mark segment-polarity gene-like expression in the pre-cheliceral region. The arrow in panel **F** points to expression in the developing eyes. Panels marked with an apostrophe (´) represent Sybr-green staining of corresponding embryos. Developmental stages are indicated after Gainett et al. [22]. Abbreviations: L4, leg four,lr, labrum; pp, pedipalp; saz, segment addition zone

We identified a single copy of *hh* in the genome and an embryonic transcriptome of the harvestman *Phalangium* [21, 81], and investigated expression of this gene in embryos ranging from stage 8 to 12 (staging after [22]). *Phalangium hh* is expressed in a typical segment-polarity gene pattern in the form of transverse segmental stripes (Fig. 5A–C, E), including a stripe in the pre-cheliceral region. *Phalangium hh* is also posteriorly expressed in the appendages (Fig. 5A, B) and in the stomodaeum (Fig. 5C-E). At later developmental stages, expression is seen in the developing eyes (Fig. 5F; cf. expression marked by an arrow with the position of the developing eyes [22, 23]).

### Expression of additional eye-markers identified in our SCS experiment: *SoxF1*, *CD36.1, optomotor-blind3, sevenless *and *engrailed-2*

**Fig. 6:**
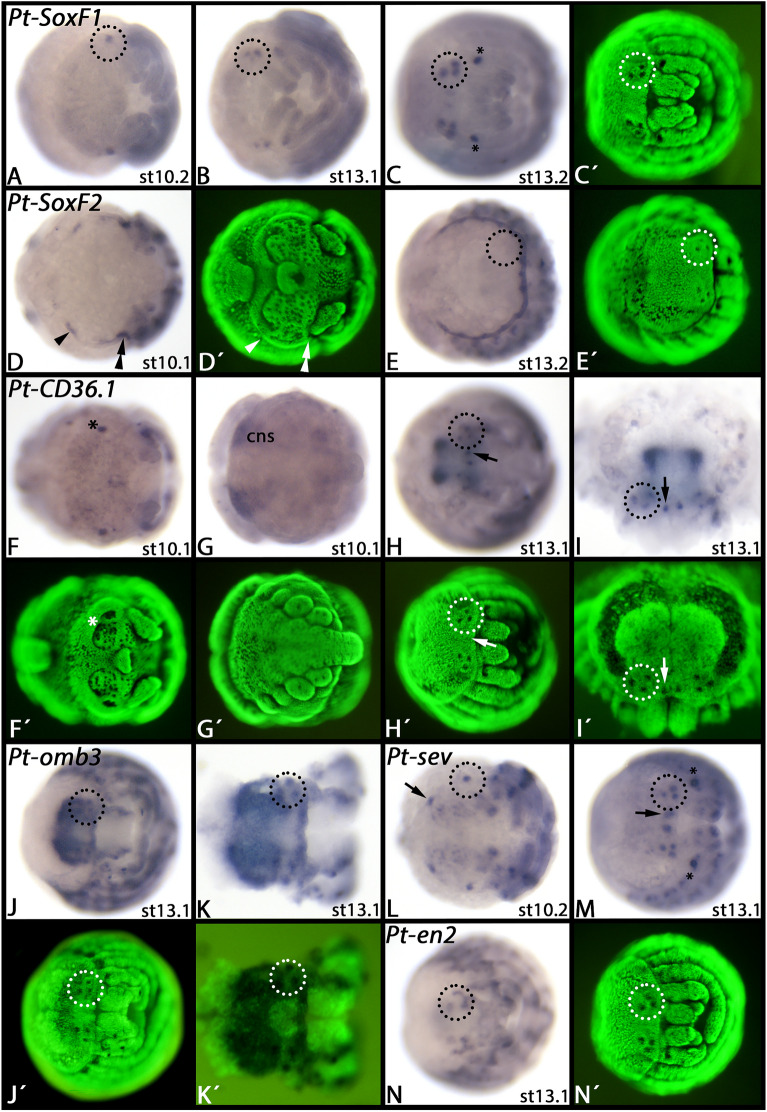
Expression of *Parasteatoda SoxF1* (panels **A**–**C**), *SoxF2* (panels **D**, **E**), *CD36.1* (panels **F**–**I**), *omb3* (panels **J**, **K**), *sev* (panels **L**, **M**), and *en2* (panel **N**). In all panels, anterior is to the left; except for panel I (anterior pointing up). Expression in the secondary eyes is encircled (dashed circles), except for panel E showing no expression in the secondary eyes. In all panels, arrows point to expression in the primary eyes. The arrowhead and double arrowhead in panel **D** point to expression that could be associated with the primordia of the secondary and primary eyes, respectively. The asterisk in panel **F** mark expression in the neurogenic ectoderm of the head lobe that is not associated with the developing eyes. The asterisks in panels **C** and **M** mark unspecific staining of the egg teeth at the dorsal base of the pedipalps. Panels marked with an apostrophe (´) represent Sybr-green staining of corresponding embryos. Developmental stages are indicated after Mittmann and Wolff [61]. Abbreviations: cns, central nervous system

Sox genes have previously been studied in *Parasteatoda*, and expression of *SoxF1* has been reported to be restricted to late developmental stages when it is expressed in the secondary eyes and the developing spinnerets ([5], their supplementary data). In addition to the previously reported pattern of *SoxF1* we found that at late stages, expression is also in the principal eyes, not only the secondary eyes (Fig. 6A–C). Interestingly, we also discovered specific expression of *SoxF2* in the anlagen of the principal and secondary eyes of *Parasteatoda* (Fig. 6D), suggesting that both genes are likely involved in eye development, and that the early and late expression of *SoxF2* and *SoxF1*, respectively, could represent another case of sub-functionalization.

*Parasteatoda CD36.1* is first expressed in a salt-and-pepper pattern in the central nervous system (Fig. 6F, G). Within the head, there are two domains of stronger expression in the neurogenic ectoderm close to the lateral furrow (Fig. 6A). Later, this tissue contributes to the developing brain that still strongly expresses *CD36.1* (Fig. 6H, I). At this point during development there are eight distinct domains of expression that clearly correlate with the six secondary and the two principal eyes (Fig. 6H, I).

The *Parasteatoda optomotor-blind3* (*omb3*) gene has also been investigated before, but expression has only been shown and described for earlier developmental stages prior to late eye-development ([55, 56]; aug3.g3790). Like other *omb* genes [36] (*omb* = *omb1*) (Supplementary File 16, panels C-K), also *omb3* is expressed in the pre-cheliceral region and in dorsal tissue of prosomal and opisthosomal appendages (Fig. 6J, K). At later developmental stages, *omb3* is expressed in a complex pattern in the developing appendages, and indeed also specifically in the developing secondary eyes, unlike its paralogs *omb1*, *omb2* and *omb4* (Fig. 6J, K and Supplementary File 16, panels C-K).

Previously published data on *sevenless* (*sev*) were not conclusive concerning expression in the developing eyes [60]. Here we show that *sev* is also expressed in the primordia of the secondary and principal eyes (Fig. 6L, M).

Likewise, previously published data on *engrailed-2* (*en2*) did not cover later developmental stages [42]. Here we show that *en2* is expressed in the six secondary eyes, but not in the two principal eyes, in late-stage embryos (Fig. 6N).

### Expression of *Parasteatodaepidermal growth factor receptor* and *glass*

**Fig. 7:**
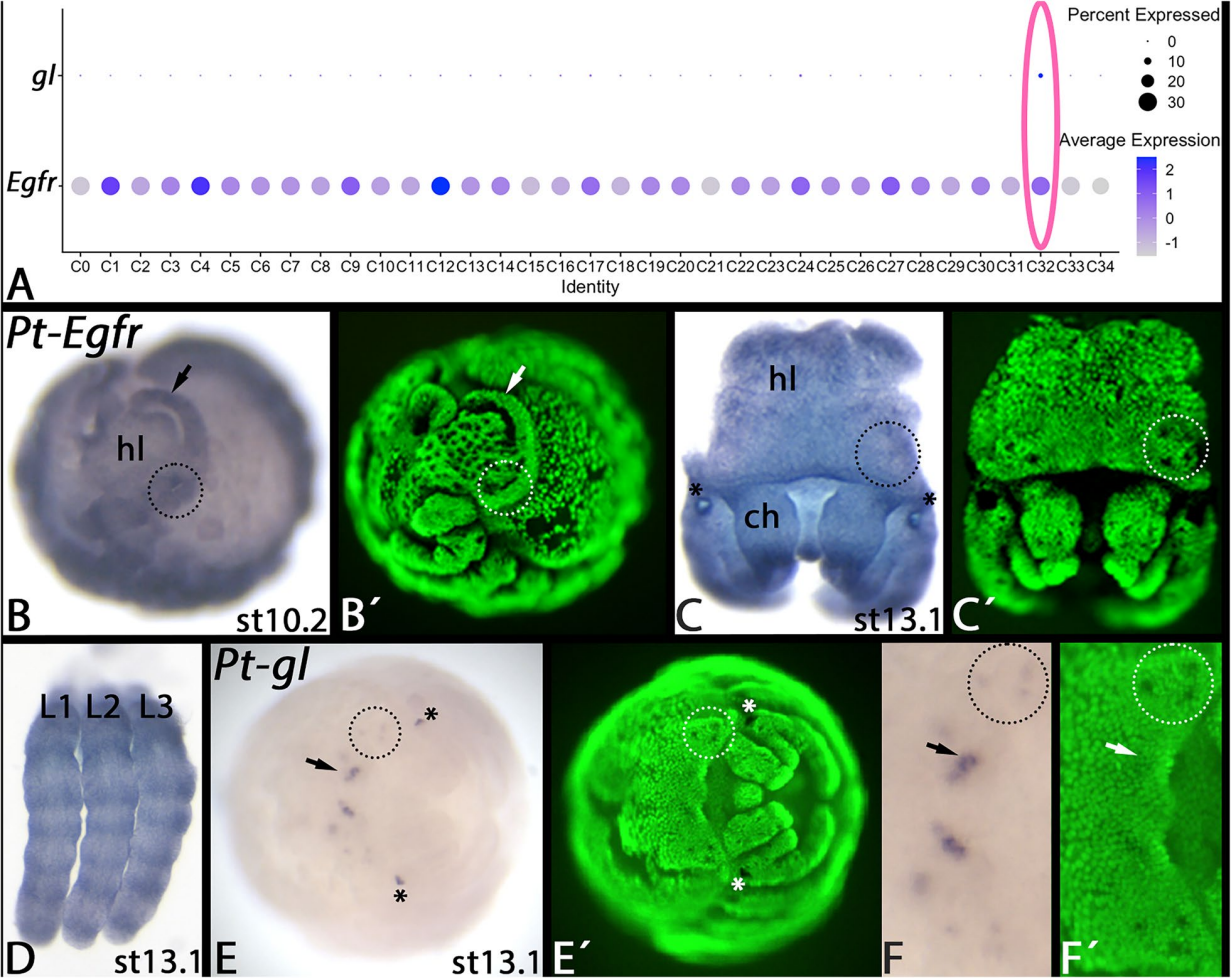
Dot plots of *gl* and *Egfr* (panel **A**), expression of *Parasteatoda Egfr* (panels **B**–**D**) and *gl* (panels **E** and **F**). The dot plots in panel **A** show the distribution of transcripts among the cells recovered in our SCS experiment. Note the ubiquitous expression of *Egfr* and the specific expression of *gl* in C32. The region of the secondary eye primordia is encircled (dashed circles) in panels **B**, **C**, **E**, and **F**. Asterisks in panels **C** and **E** mark unspecific signal in the egg teeth. The arrows in panels **E** and **F** point to expression in the primordium of the primary eyes. Panel **F** shows a magnification of the embryo shown in panel **E**. Panels marked with an apostrophe (´) represent Sybr-green staining of corresponding embryos. Developmental stages are indicated after Mittmann and Wolff [61]. Abbreviations: ch, chelicera; hl, head lobe. L1-L3, first to third leg

Epidermal growth factor receptor (*Egfr*) and *glass* (*gl*) play important roles during eye development in the fly *Drosophila* (reviewed in [18]). Although these two crucial factors were not among the enriched marker genes in the C32 cluster, we found the strongest expression of *gl* in a subset of C32 and *Egfr* was moderately expressed in all cell types (Fig. 7A). Therefore, we also investigated the embryonic expression to test whether they may play conserved roles in spider eye development as well.

We find that *Egfr* indeed is expressed ubiquitously, but is upregulated in certain tissues, including the primordia of the primary and secondary eyes (Fig. 7A-C). Interestingly, at later developmental stages, when the indentations of the secondary eyes have formed, *Egfr* appears to be specifically downregulated in these areas (Fig. 7C). Such downregulation is not seen in the area where the primary eyes form. Another interesting aspect of *Egfr* is its expression in the form of rings in the developing appendages that suggest a function in joint formation (Fig. 7D).

Expression of *gl* is restricted to later developmental stages and is weakly recognizable in the secondary eyes, but strongly in the primary eyes (Fig. 7A, E, F).

## Discussion

### The role of *hh* in spider and panarthropod eye development

The *hh1* gene of spiders is expressed in the form of a transverse stripe in the pre-cheliceral region which is part of the head lobes. This domain, however, does not cover the primordia of the secondary eyes (Supplementary File 15). Baudouin-Gonzalez et al. [6] suggested that *hh1* (*hh* in their paper) could aid Wnt-signalling in limiting the optical field and thus restrict eye growth/size. In any case, the expression of spider *hh1* clearly shows that this gene cannot be involved in retina development and differentiation as it is the case in *Drosophila* (reviewed in e.g. [18]). In *Drosophila*, *hh* is activated by So (e.g. [74]), and subsequently, expression of *ato* is dependent on Hh-signalling (e.g. [14, 27]) making Hh-signalling a crucial component of eye development in the fly. The apparent lack of *hh1* expression in the developing eyes of spiders raised the question if this crucial function of *hh* could have been gained somewhere in the lineage leading to *Drosophila*, or if it could have been lost in the lineage leading to spiders. Both scenarios would imply substantial differences in the eye-developmental gene regulatory network in either of these two groups of arthropods.

The finding of a second paralog of *hh* in spiders (i.e. *hh2*) answers this question in a very satisfying way, showing that Hh-signalling (as inferred by the expression of its ligand encoding gene, *hh2*) is indeed involved in eye development in both the fly and spiders. The relatively late onset of *hh2* expression in the developing eyes is in line with a dependence on *so* as it also is the case in *Drosophila* (e.g. [71]; cf. [7]). The most likely scenario in spiders is that the two copies of *hh* have undergone sub-functionalization after a duplication that occurred in the lineage leading to Arachnopulmonata (i.e. scorpions, whip spiders, and spiders) (e.g. [82]), and indeed similar cases of sub-functionalization in spiders have been recorded frequently (e.g. [1, 41, 55, 80]).

Despite the fact that *hh* has been the subject of various studies in various groups of arthropods including other chelicerates, myriapods, crustaceans and insects beyond *Drosophila* (e.g. [16, 32, 35, 37, 62, 84]), data on the potential role of *hh* in eye development are scarce. In the scorpion *Euscorpius flavicaudis*, for example, expression has only been reported for one paralog of *hh* and during stages that reflect germ band extension [84]. The investigated *hh* gene, however, is expressed in the semi-lunar grooves at late germ band stages, and this is where the eyes of the scorpion will develop [58, 84]). In the myriapod *Glomeris marginata*, the single *hh* ortholog is expressed in exactly the place where the eyes will form ([35], their Fig. 6G; cf. [75] for expression of *Pax6* as marker of *Glomeris* eye-primordia). In insects other than *Drosophila*, *hh* is at least expressed anteriorly in the head lobes during germ band formation and extension [4, 62], but again conclusive data on a potential function of *hh* in eye development in these species are missing. As we show in this paper, expression of *hh* in the harvestman *Phalangium* is very similar to that in other arthropods, including expression in the developing eyes. We thus conclude that this expression, including expression in the eyes, is ancestral and covered by the two paralogs in spiders.

In the outgroups, tardigrades and onychophorans, *hh* expression has only been studied in the closely related velvet worms *Euperipatoides kanangrensis* [38] and *Euperipatoides rowelli* [17], but with a focus on body axis segmentation. A closer look at the expression of *hh* in *Euperipatoides kanangrensis*, however, revealed no expression in the developing eyes, at least not prior to and including stage 20 [38]. Due to the lack of further comparative data, especially those from tardigrades, it thus remains unclear if the role of *hh* in eye development originated in the lineage leading to arthropods, the lineage leading to panarthropods, or even earlier, representing a case of deep homology (cf. data on *hh* function in vertebrates (e.g. [68, 83]).

### Primary eyes of spiders with or without *hh2* expression?

According to our data, expression of *hh2* in the developing eyes is possibly not fully conserved in different groups of spiders, at least with respect to expression in the primary eyes (Fig. 4). The detected pattern, however, is not reflecting spider phylogeny [49] because we see expression in the primary eyes of the entelegyne RTA-class spider *Pardosa*, not in the more basally branching entelegyne spider *Parasteatoda*, but in the most basally branching synspermiatan spider *Pholcus,* and the mygalomorph spider *Ischnothele* (Fig. 4). This could mean independent recruitment of *hh2* in the development of the primary eyes in the lineages leading to *Ischnothele*, *Pholcus* and *Pardosa*, or more likely a secondary loss in the lineage leading to *Parasteatoda* or failure to detect *hh2* in *Parasteatoda* due to heterochrony (delayed onset of *hh2* expression in the primary eyes). In this context, it may be interesting to mention that we cannot detect *en2*, the putative activator of *hh2*, in the primary eyes of *Parasteatoda* either [42] (Fig. 6N).

A very recent study reported differences in the expression of some RDGs in the different eye types of spiders suggesting that these differences may indeed be correlated with different eye types, specification/specialization of eye types, and consequently ecological adaptation of spiders [7]. Similar differences were previously reported for *Pax6* genes in spiders (cf. [77, 79]). The differences seen in *hh2* expression in the principal eyes of some spiders and the lack in others may thus contribute to the proposed differentiation, specialization and ecological adaptation reported previously.

### Hypothesized ancient interactions in visual sense organ development

**Fig. 8:**
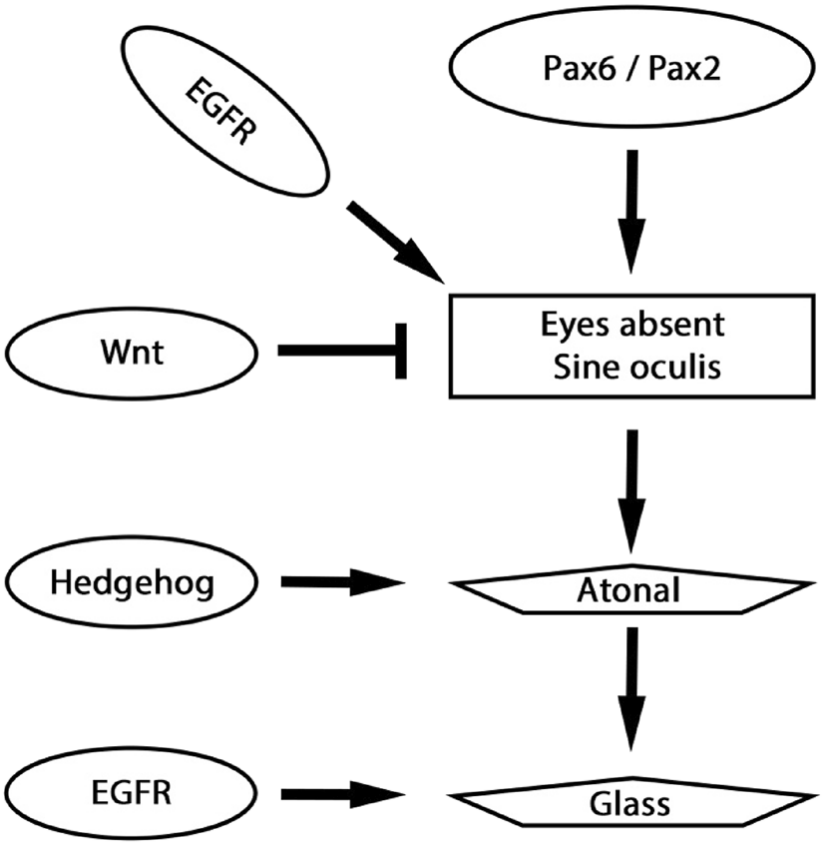
Hypothesized core of the ancestral gene regulatory network of visual sense organ development in arthropods. Combined data from Friedrich [18], Janeschik et al. [34], Baudouin-Gonzales et al. [6], and this study. Available data suggest that Pax genes *Pax6* and/or *Pax2* and Egfr-signalling activate the expression of *eya* and *so*. Likewise, the data suggest that Wnt-signalling restricts the area of *eya* and *so* activity, that Eya, So, and Hh-signalling activate *ato*, and that Ato and Egfr-signalling activate *gl*. Note that many of the data are based on gene expression analysis, and that additional functional data are needed to verify the proposed gene interaction

In one of his review papers on *Drosophila* eye development, Friedrich [18] suggested a core genetic program that mediates visual sense organ (eye) development, because the factors involved in this network represent key players of all visual sense organs in the fly, i.e. the compound eyes, the ocelli, and the Bolwig organs (present in the larvae). Here, *Pax6* is seen as the direct activator of *eya* and *so*, *dpp* as an activator of *eya*, and *wg* (or Wnt-signalling in general) as an inhibitor of *eya* and *so* in optic field specification. *eya* and *so*, together with *hh*, then activate *ato,* and *ato* together with epidermal growth factor receptor (Egfr)-signalling activates *gl*, the final target of this GRN [18] (Fig. 8).

In this scenario, *hh* is thus a conserved ancient member of the GRN that regulates eye development. From top to bottom of this GRN and in the “eyes” of a spider, firstly *Pax6* is not expressed in all developing eyes [7, 77, 79]. A recent study, however, revealed that another Pax gene, *Pax2* could substitute for *Pax6* [19, 34]). *eya* and *so* are both expressed in the primordia of all eyes in spiders [7, 79], and this is where Wnt-signalling mostly does not take place [6, 41]. Expression of *dpp* in or near the developing eyes, however, could not be detected raising the question if this component of the suggested GRN may not be part of the corresponding GRN in spiders [6]. The combined expression data from several groups of spiders presented in this paper, however, suggest that Hh-signalling, as represented by the expression of *hh2* contributes to the development of all eyes (Fig. 4), and as predicted by Friedrich [18], also *ato* is expressed in all eyes of spiders [6, 7]. The expression of *Egfr* appears to contradict a specific function in spider eye development because it is downregulated in the region where the secondary eyes form. Interestingly, however, it has been reported previously that *Egfr* mRNA is downregulated in certain tissues of *Drosophila* as the result of elevated Egfr-signalling in these tissues [88]. The specific downregulation of *Egfr* mRNA in the secondary eye primordia (Fig. 7C) could thus be the result of preceding strong Egfr-signalling (Fig. 7B). The gene expression analysis is thus in line with a conserved role of Egfr-signalling and Ato in the regulation of the terminal gene *gl* which also is expressed in the primordia of all spider eyes (Fig. 7E, F). The combined data on spider eye development, including our new data on *hh2*, initially identified in our SCS data set, *Egfr*, and *gl* thus support the scenario that Friedrich [18] suggested about a conserved ancestral eye-developmental GRN (Fig. 8). Surprisingly, this network appears to be conserved in all eyes of spiders but has been modified in the eyes of flies. The eyes of spiders thus appear to represent less-derived optical organs, at least when compared to insects, making spider eyes interesting targets for further investigation and the understanding of eye evolution in arthropods and animals in general.

### *SoxF*, a new player in spider eye development

*SoxF* genes are not expressed during panarthropod eye development [40], except for the previously reported expression of *SoxF1* in the primordia of the developing secondary eyes of *Parasteatoda* [5]. Expression and function of *Parasteatoda SoxF1* thus clearly represents a case of neo-functionalization and identifies *SoxF1* as a new player in the concert of spider eye development. From the reported data, it appeared tempting to speculate that *SoxF1* is a unique new factor of secondary eye development [5]. We, however, also detected faint expression of *SoxF1* in the principal eyes of *Parasteatoda* (Fig. 6C). We conclude that *SoxF1* is not a specific factor of secondary eye development, but rather a general new factor of spider eye development. The expression of *SoxF2* (Fig. 6D) is in line with an earlier function of this gene in the primordia of both the secondary and principal eyes of the spider (cf. expression of *so1* in *Parasteatoda* [79]), and thus *SoxF* orthologs could have been the target of temporal sub-functionalization in arachnopulmonates, or at least spiders, after the recruitment as eye-developmental factors. The remaining question is when *SoxF* has been recruited as an eye-developmental gene. Due to the lack of comparative data from other spiders, and indeed other chelicerates, this question needs to be addressed in the future.

### *CD36.1*, a spider CD36-family gene that is expressed in the developing eyes

The CD36 gene family comprises four orthologs in mammals, six in the nematode worm *Caenorhabditis elegans*, two in an onychophoran, five in a tick (Chelicerata), eight in a myriapod, six to eight in crustaceans, and between 12 and 14 in insects [9, 29, 66, 93]. These genes fulfil a plethora of functions including cytoadhesion, carotenoid transport, and chemoreception. Many of these genes have not been investigated in *Drosophila*, other insects, or indeed any other (pan) arthropod species rendering comparative analysis difficult. At least in *Drosophila*, however, comprehensive gene expression studies and functional analysis have identified some specific functions of some members (summarized in [29]). Among them are two genes, *NinaD* and *santa-maria*, that are involved in the redistribution of carotenoids to the developing eyes (*NinaD*; [94]) and the biosynthesis of rhodopsin in neurons and glia cells outside the retina (*santa-maria*; [95]). The expression pattern of *santa-maria* in the ventral nerve cord, the brain, and the developing eyes is at least superficially similar to that of the *Parasteatoda CD36.1* gene. This could suggest a conserved function of arthropod Group-2 CD36-family genes in arthropod nervous system and eye development. Addressing this question, however, would at least require a comprehensive analysis of arthropod CD36-family genes and accompanying gene expression analyses.

## Conclusions

In this paper we show that single-cell sequencing (SCS) of developing embryos can reveal specific tissue- and structure-specific new and unexpected genes, and thus can provide insight into correlated gene regulatory networks (GRNs) beyond the classical candidate gene approach. SCS will thus undoubtedly help untangling complex GRNs in model and non-model organisms, especially with respect to differences between those new models and the well-studied established models such as the fly *Drosophila*, the mouse *Mus* and the nematode worm *Caenorhabditis*. Once identified, the evolutionary origin of new molecular players (genes) involved in the development of morphological structures can be tested by means of comparative studies as exemplified in this study for the *hh2* paralog in different groups of spiders.

## Supplementary Information

Additional file 1: Gene expression matrix (zipped folder)

Additional file 2: Spreadsheet Markers per cluster and *Drosophila* annotation (EXCEL file)

Additional file 3: Seurat_Object (zipped folder)

Additional file 4: Hh_Alignment (.tif file)

Additional file 5: Hh_Nexus

Additional file 6: Identifiers_Primers_EyeClusterGenes (EXCEL file)

Additional file 7: CD36_Alignment (.tif file)

Additional file 8: CD36_Nexus

Additional file 9: Phylogenetic tree of CD36-family genes. Bayesian analysis using MrBayes applying 0.5 million cycles for the Metropolis-Coupled Markov Chain Monte Carlo (MCMCMC). The tree is midpoint rooted. Node labels represent posterior possibilities. The scale bar represents 0.3 amino acid substitutions per site. The identified *CD36.1* gene is marked with a red asterisk. Note that this gene appears to possess a second paralog in the spider (XP021004437.1). The sequence of *Parasteatoda CD36.1*. Is most similar to that of *Drosophila* Group2A and B genes but does not represent a one-to-one ortholog of either of these six *Drosophila* CD36-family genes (see text for further information). Species abbreviations: Dm, *Drosophila melanogaster*; Dr, *Danio rerio*; Mm, *Mus musculus*; Pt, *Parasteatoda tepidariorum*

Additional file 10: Additional gene expression data. Expression of *unc0466* (A-D), *opa* (E-H), *lim1* (I, J), *lim2* (K, L), *unc4232* (M, N), *scro* (O, P), *unc3221* (Q), *unc7000* (R), *gsc* (S), *mmr1* (T, U), *mmr2* (V), and *FMRFa-R* (W). In all panels, anterior is to the left, except panel W (dissected leg, dorsal to the left; distal end pointing downwards). Arrows in panel W point to expression inside the leg. Abbreviations: aSp, anterior spinneret; bl, book lung; ch, chelicera; h, heart; hl, head lobes; L, leg; pSp, posterior spinneret; s, stomodaeum.

Additional file 11: GO analysis results list (EXCEL file)

Additional file 12: Info_number_orthologs_per_cluster (WORD file: Table)

Additional file 13: GO analysis results barplots (zipped folder)

Additional file 14: Feature plots of all markers of cluster C32 (zipped folder)

Additional file 15: Expression of spider *hh1* genes. In all panels, anterior is to the left. Panels A, C, D, F, G, H, L, M, N, O and R represent lateral views. Panels B, E, I, J, P and Q represent lateral views (in these panels, dorsal is up). Panel K represents a dorsal view. Note the segment-polarity gene-like expression of all *hh1*-group genes, expression along the appendages, expression in the segment addition zone (saz), and expression in the anlagen of the stomodaeum (asterisks in all panels). The arrow in panel O points to four dots of expression in the neuronal ectoderm that thus are not associated with the developing eyes. In all panels, full circles mark expression in the pre-cheliceral region, but note that this expression is not at the place where the eyes will form. Panels B´, H´, I´, L´, O´ and P´ represent Sybr-green staining of corresponding embryos. Developmental stages are indicated after Mittmann and Wolff [61]; note that developmental stages in different species of spiders have been defined by comparable morphological landmarks such as the overall shape of the embryo and the length of the appendages. Abbreviations: ch, cheliceral segment; L, leg-bearing segment; pp, pedipalp-bearing segment; saz, segment addition zone.

Additional file 16: Expression of *Parasteatoda* *hh2-r* (panels A and B), *omb2* (panels C-E), *omb3* (panels F-I), and *omb4* (panels J and K). In all panels, anterior is to the left. The asterisks in panels C, D, and F-H mark expression in the developing labrum. The primordia of the secondary eyes are encircled in one half of the head lobes in panels D, E, J, and K. Note that there is no expression of *omb2* and *omb4*. The dot-like expression seen in panels A and B are in the yolk and the dorsal field, but not in the embryo proper. Abbreviations: hl, head lobe; saz, segment addition zone.

## Data Availability

The datasets supporting the conclusions of this article are included within the article (and its additional files).
