# Supplementary figures and images for: Single-cell sequencing suggests a conserved function of Hedgehog-signalling in spider eye development
Source: EvoDevo. 2024 Sep 26;15:11. doi: 10.1186/s13227-024-00230-6 (PMC11428483; doi:10.1186/s13227-024-00230-6)

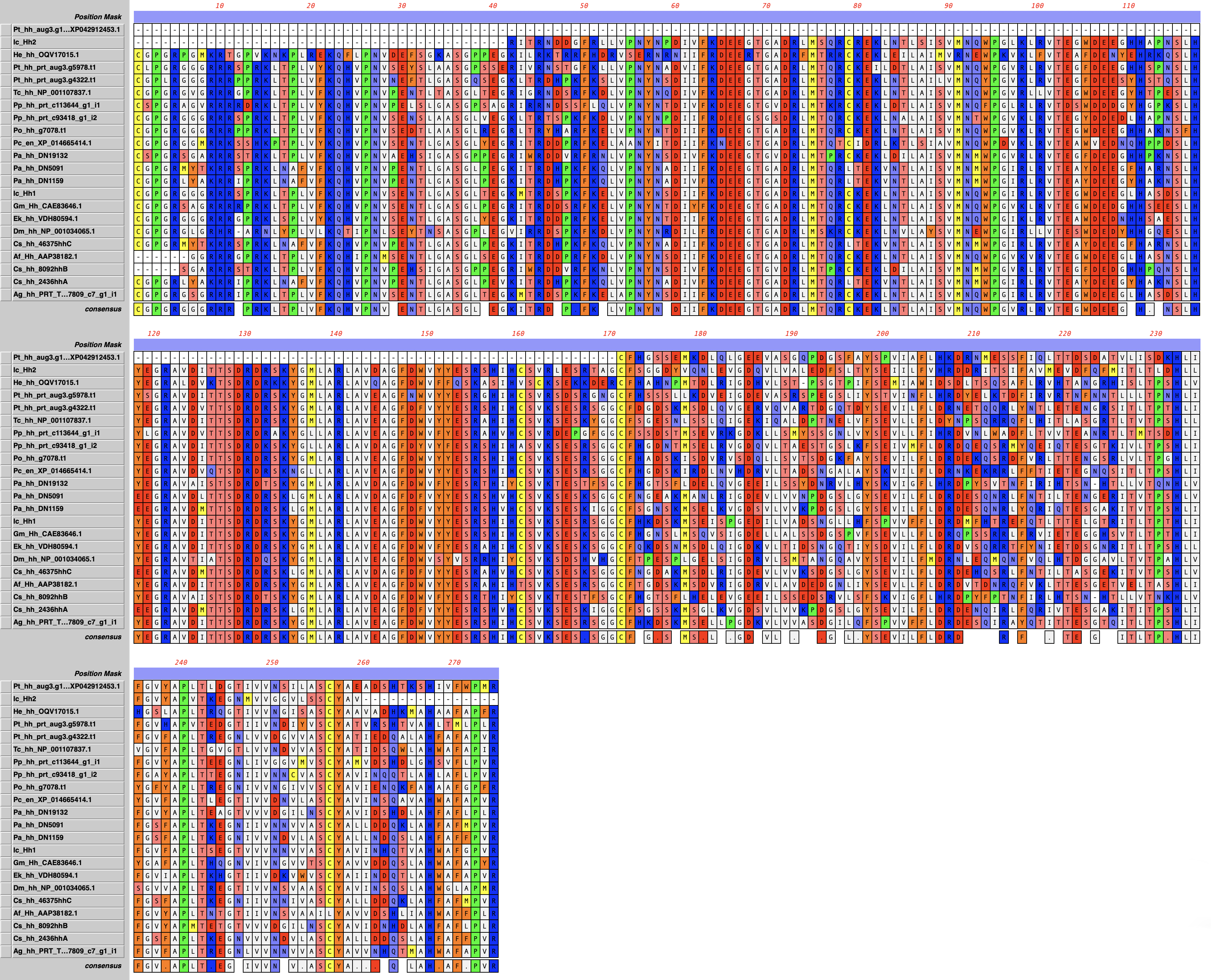

Supplement: Supplementary file 4 — Additional file 4: Hh_Alignment (.tif file) [file 13227_2024_230_MOESM4_ESM.tif]

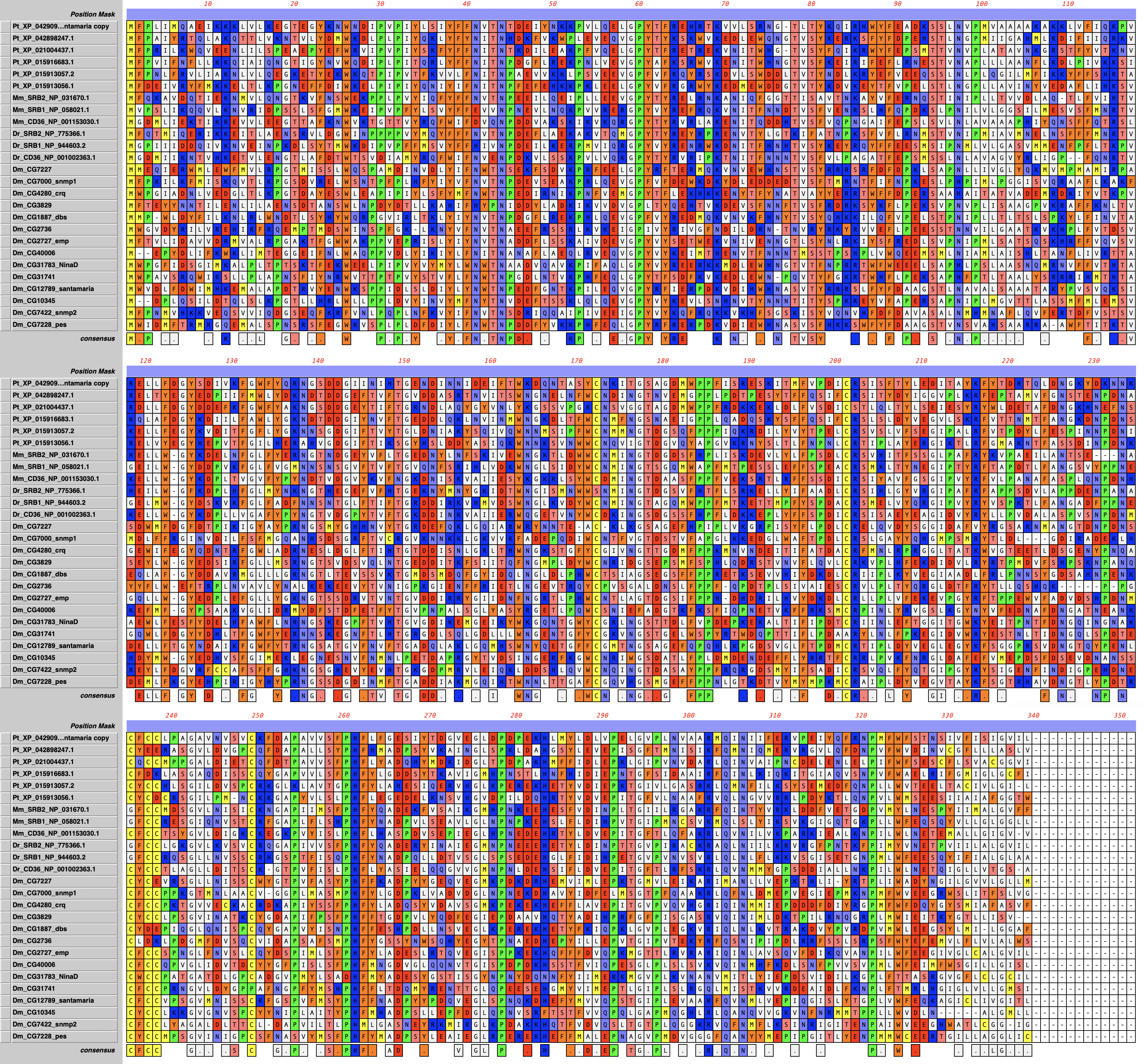

Supplement: Supplementary file 7 — Additional file 7: CD36_Alignment (.tif file) [file 13227_2024_230_MOESM7_ESM.tif]

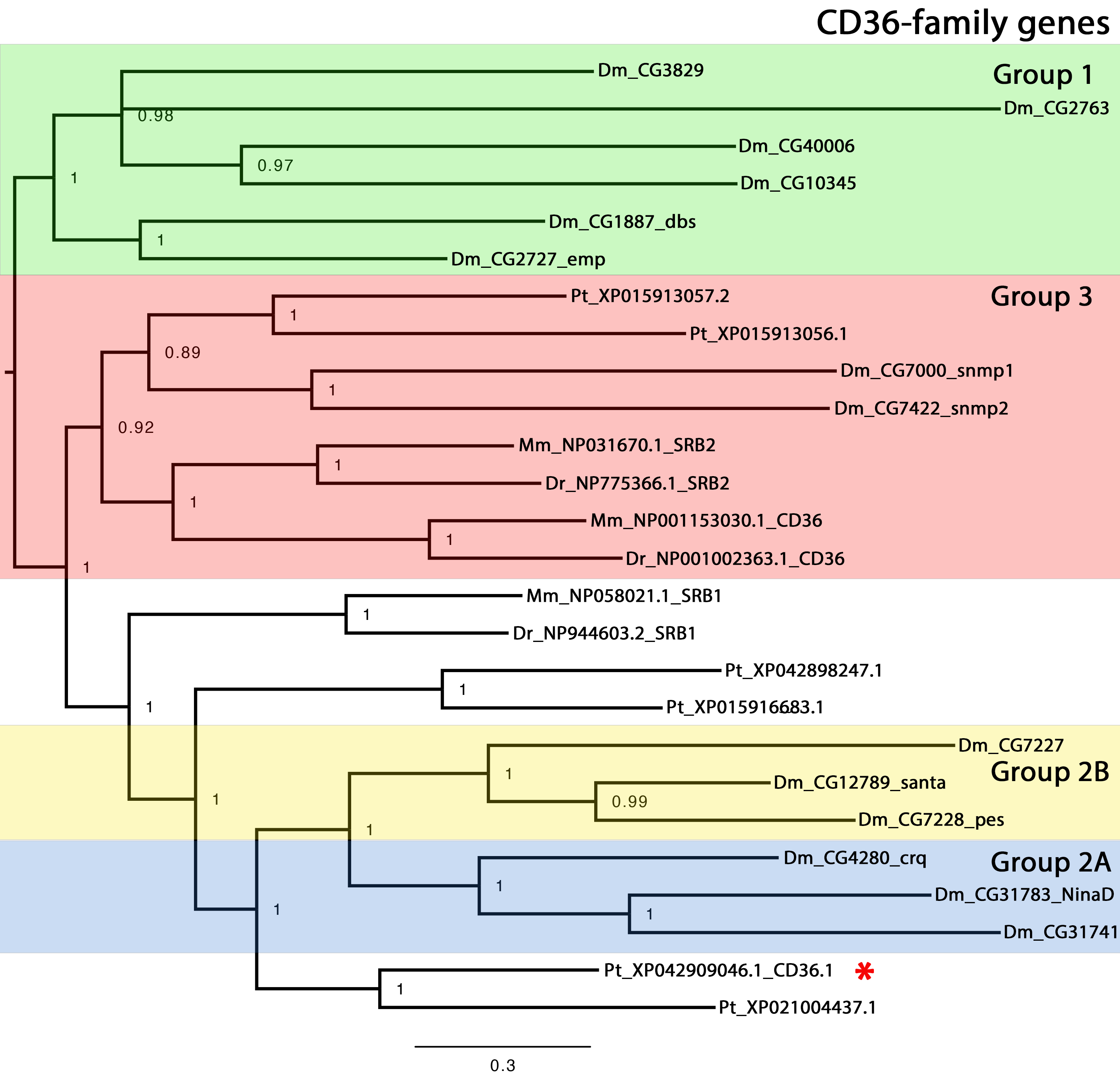

Supplement: Supplementary file 9 — Additional file 9: Phylogenetic tree of CD36-family genes. Bayesian analysis using MrBayes applying 0.5 million cycles for the Metropolis-Coupled Markov Chain Monte Carlo (MCMCMC). The tree is midpoint rooted. Node labels represent posterior possibilities. The scale bar represents 0.3 amino acid substitutions per site. The identified CD36.1 gene is marked with a red asterisk. Note that this gene appears to possess a second paralog in the spider (XP021004437.1). The sequence of Parasteatoda CD36.1. Is most similar to that of Drosophila Group2A and B genes but does not represent a one-to-one ortholog of either of these six Drosophila CD36-family genes (see text for further information). Species abbreviations: Dm, Drosophila melanogaster; Dr, Danio rerio; Mm, Mus musculus; Pt, Parasteatoda tepidariorum [file 13227_2024_230_MOESM9_ESM.tif]

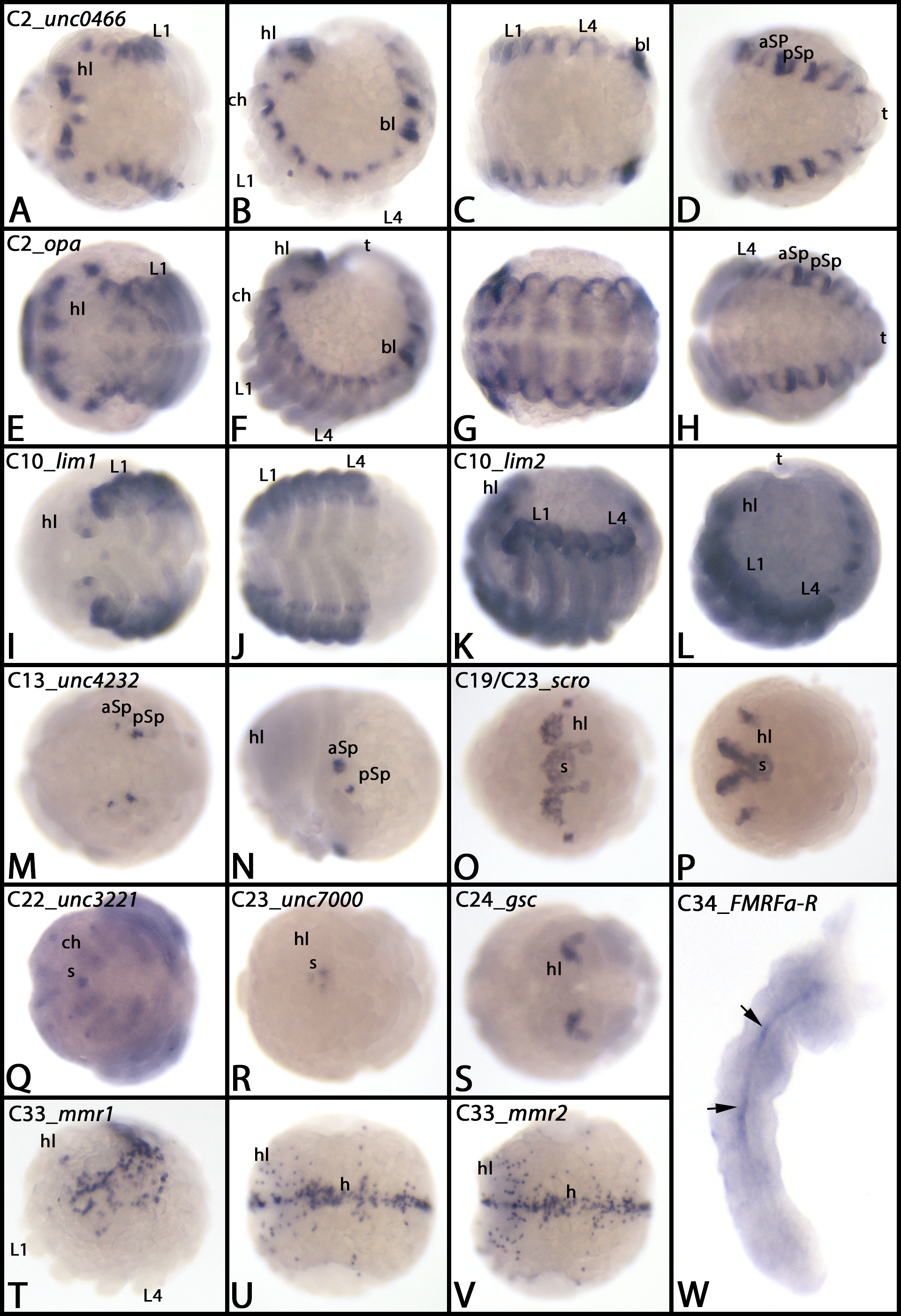

Supplement: Supplementary file 10 — Additional file 10: Additional gene expression data. Expression of unc0466 (A-D), opa (E-H), lim1 (I, J), lim2 (K, L), unc4232 (M, N), scro (O, P), unc3221 (Q), unc7000 (R), gsc (S), mmr1 (T, U), mmr2 (V), and FMRFa-R (W). In all panels, anterior is to the left, except panel W (dissected leg, dorsal to the left; distal end pointing downwards). Arrows in panel W point to expression inside the leg. Abbreviations: aSp, anterior spinneret; bl, book lung; ch, chelicera; h, heart; hl, head lobes; L, leg; pSp, posterior spinneret; s, stomodaeum. [file 13227_2024_230_MOESM10_ESM.tif]

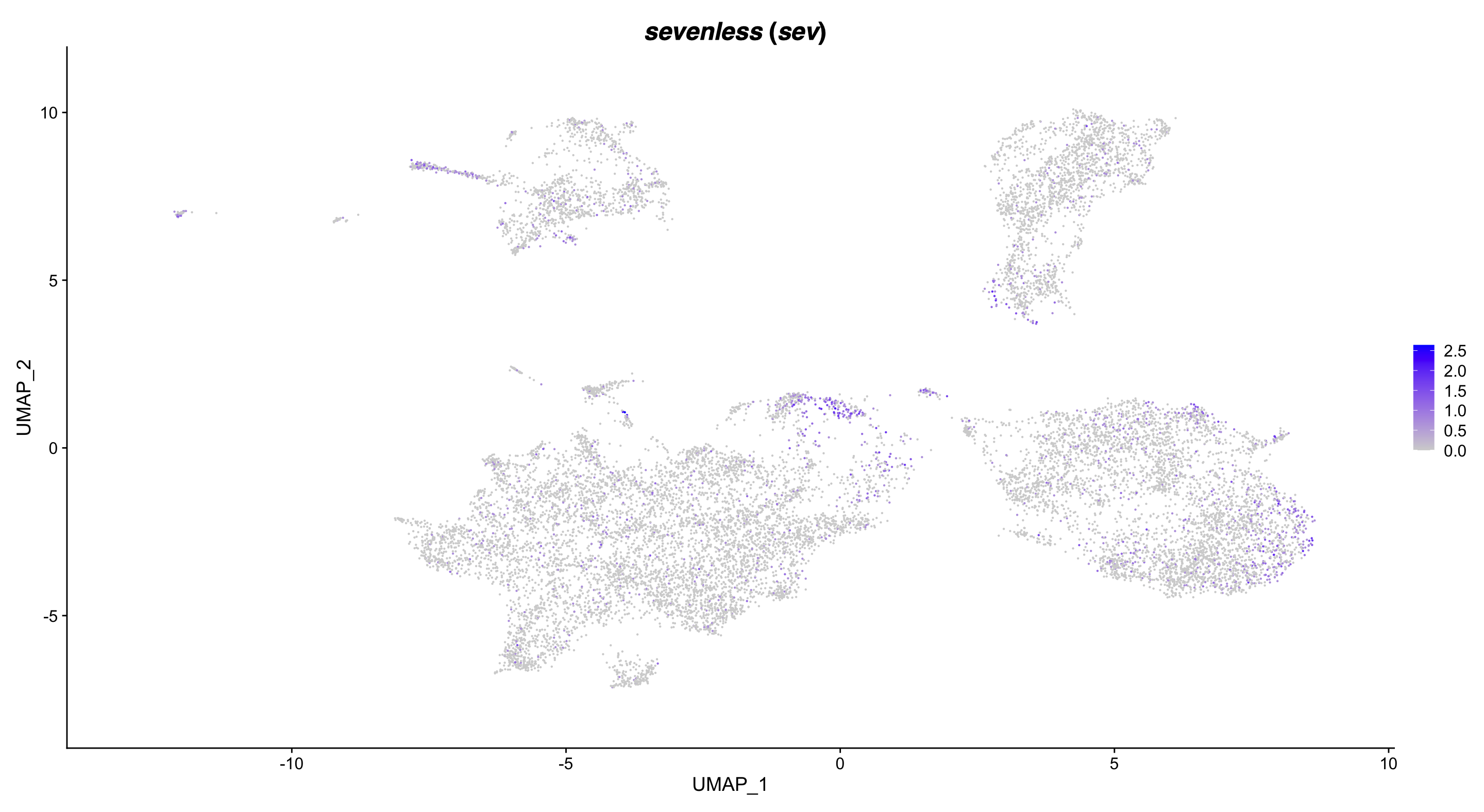

Supplement: Supplementary file 13 — Additional file 13: GO analysis results barplots (zipped folder) [file 13227_2024_230_MOESM13_ESM.zip › Supplementary File 14 - feature plots of C32 markers/19.Pt-sev LOC107441450.png]

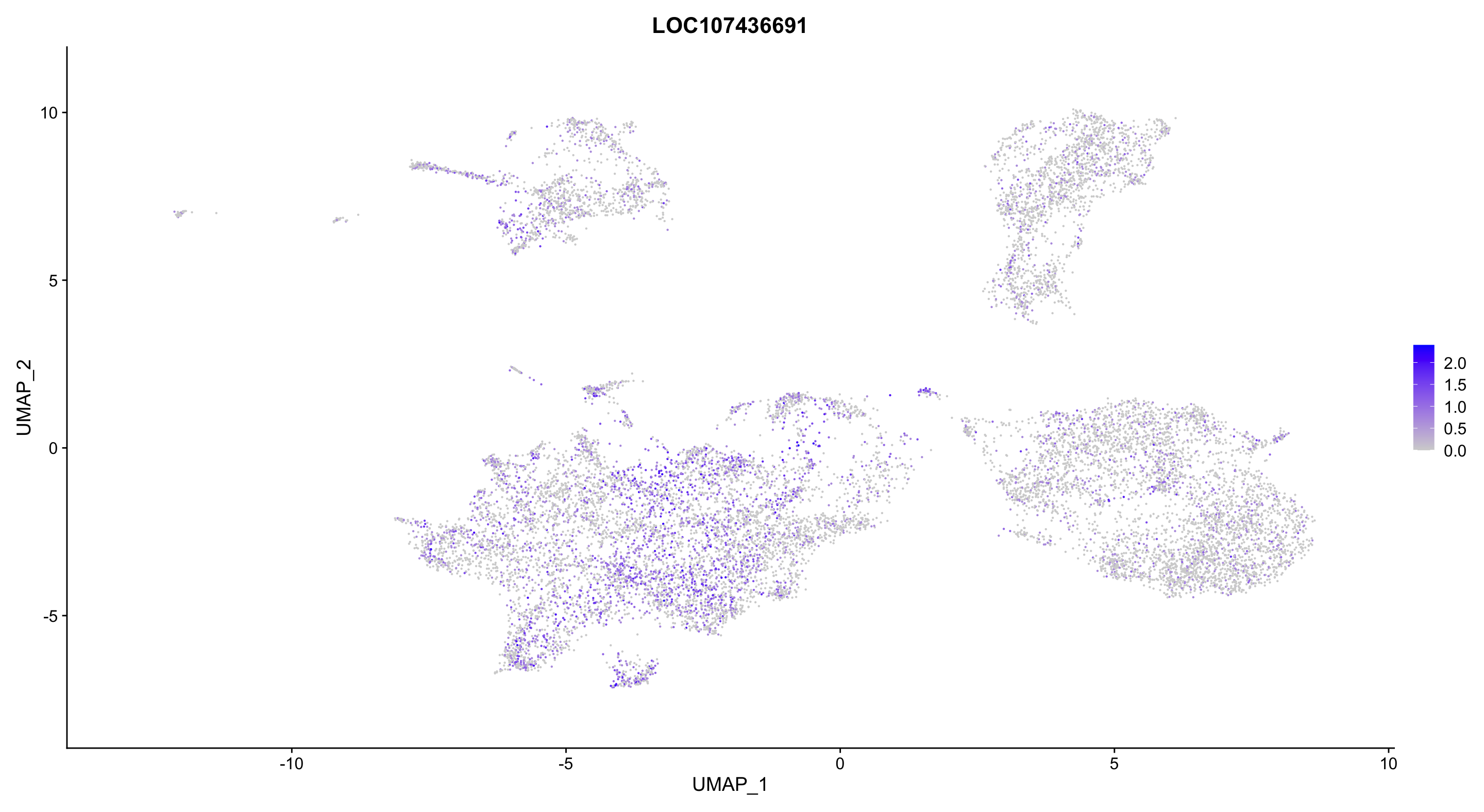

Supplement: Supplementary file 13 — Additional file 13: GO analysis results barplots (zipped folder) [file 13227_2024_230_MOESM13_ESM.zip › Supplementary File 14 - feature plots of C32 markers/25.Pt-unc6691 LOC107436691.png]

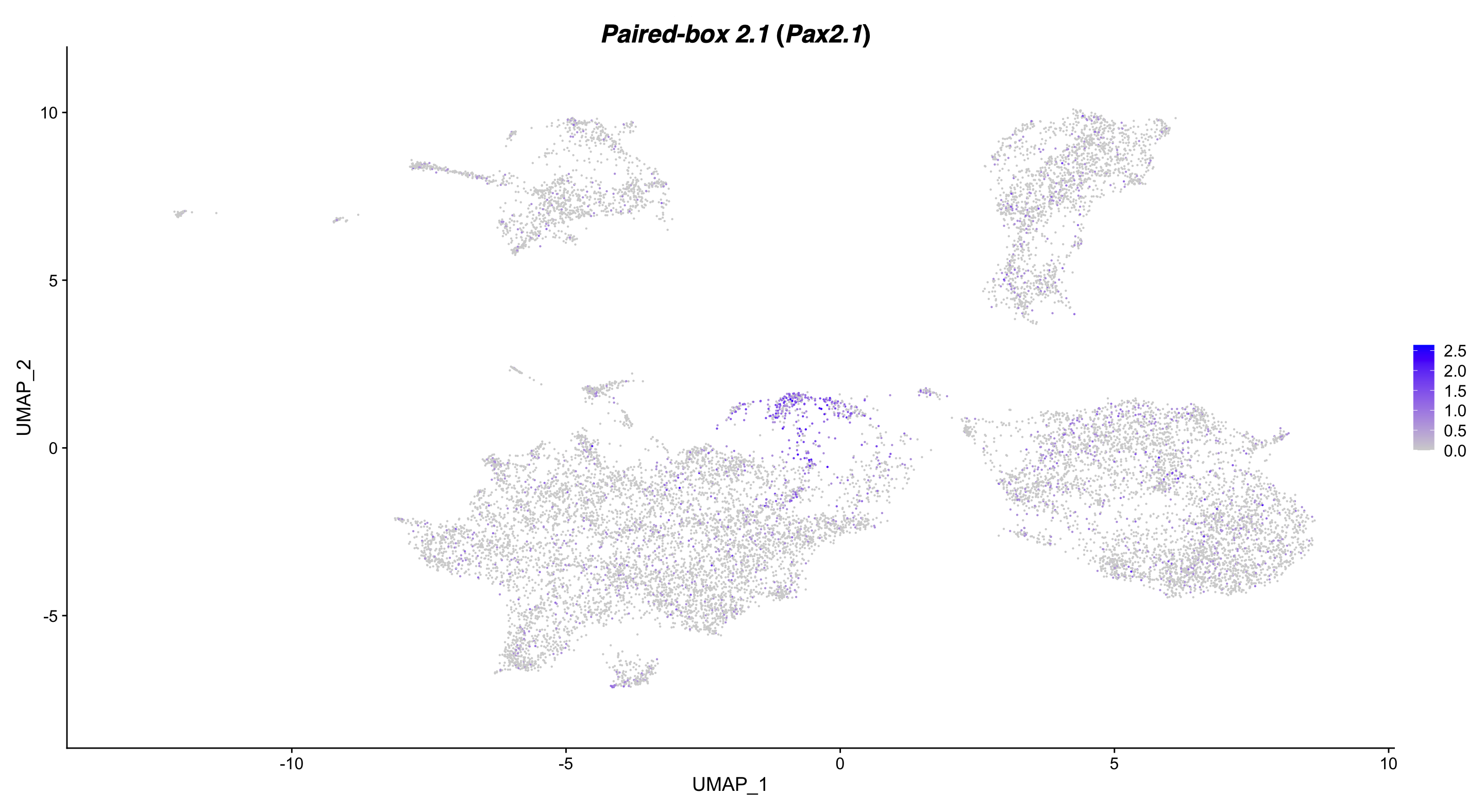

Supplement: Supplementary file 13 — Additional file 13: GO analysis results barplots (zipped folder) [file 13227_2024_230_MOESM13_ESM.zip › Supplementary File 14 - feature plots of C32 markers/21.Pt-Pax2.1 LOC107444558.png]

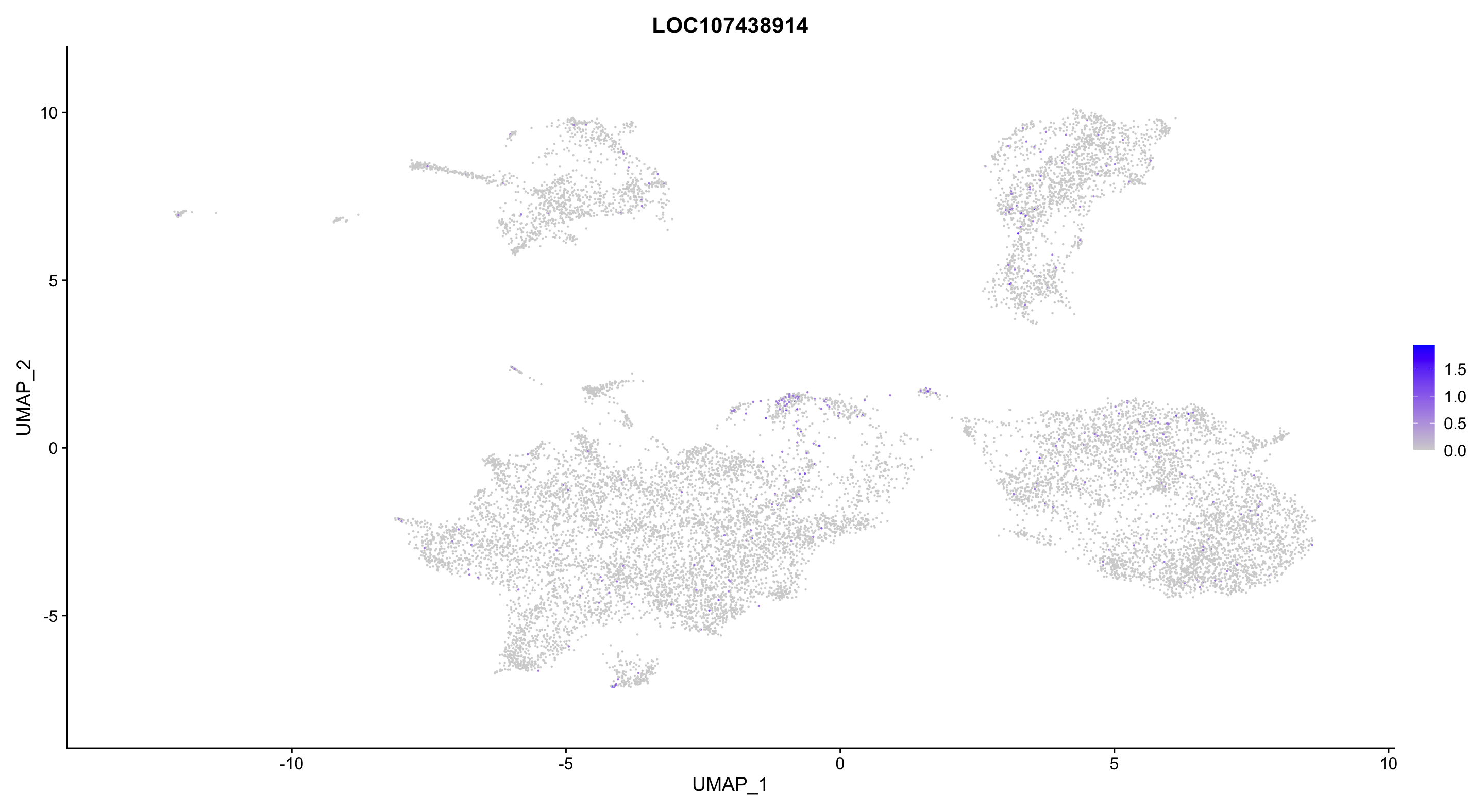

Supplement: Supplementary file 13 — Additional file 13: GO analysis results barplots (zipped folder) [file 13227_2024_230_MOESM13_ESM.zip › Supplementary File 14 - feature plots of C32 markers/10.Pt-unc8914 LOC107438914.png]

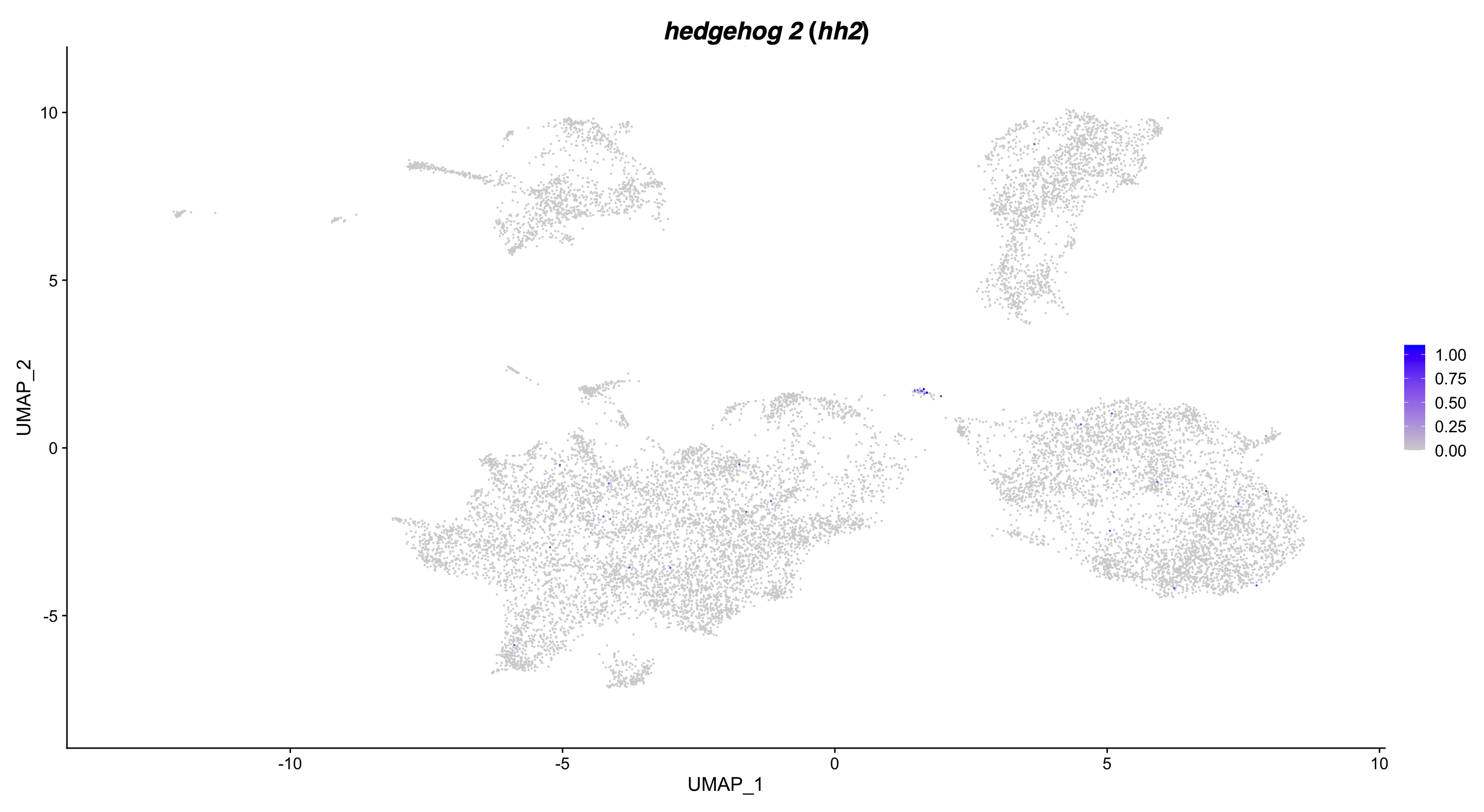

Supplement: Supplementary file 13 — Additional file 13: GO analysis results barplots (zipped folder) [file 13227_2024_230_MOESM13_ESM.zip › Supplementary File 14 - feature plots of C32 markers/1.Pt-hh2 LOC107454029.png]

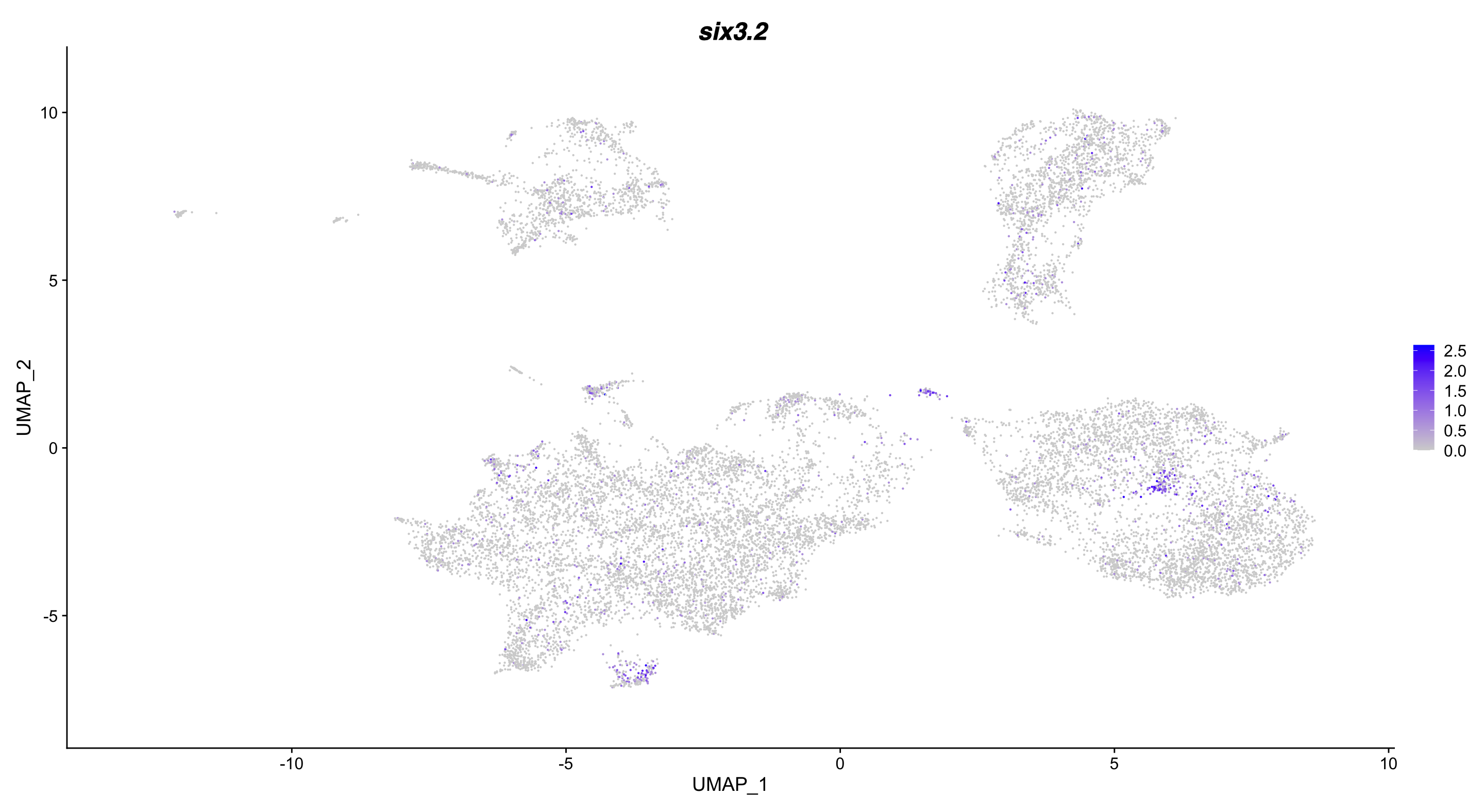

Supplement: Supplementary file 13 — Additional file 13: GO analysis results barplots (zipped folder) [file 13227_2024_230_MOESM13_ESM.zip › Supplementary File 14 - feature plots of C32 markers/3.Pt-six3.2 LOC107436457.png]

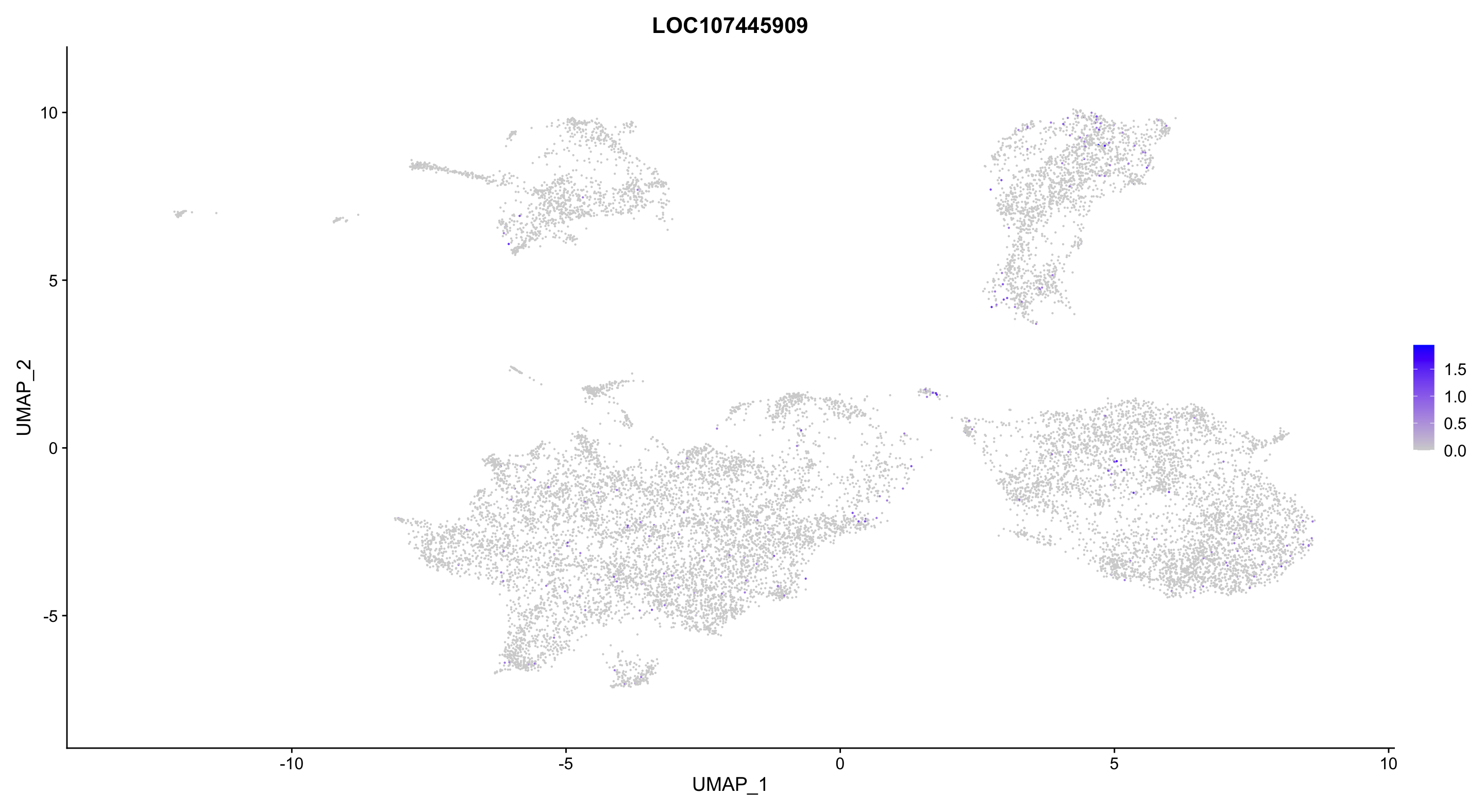

Supplement: Supplementary file 13 — Additional file 13: GO analysis results barplots (zipped folder) [file 13227_2024_230_MOESM13_ESM.zip › Supplementary File 14 - feature plots of C32 markers/13.Pt-CG31637-like LOC107445909.png]

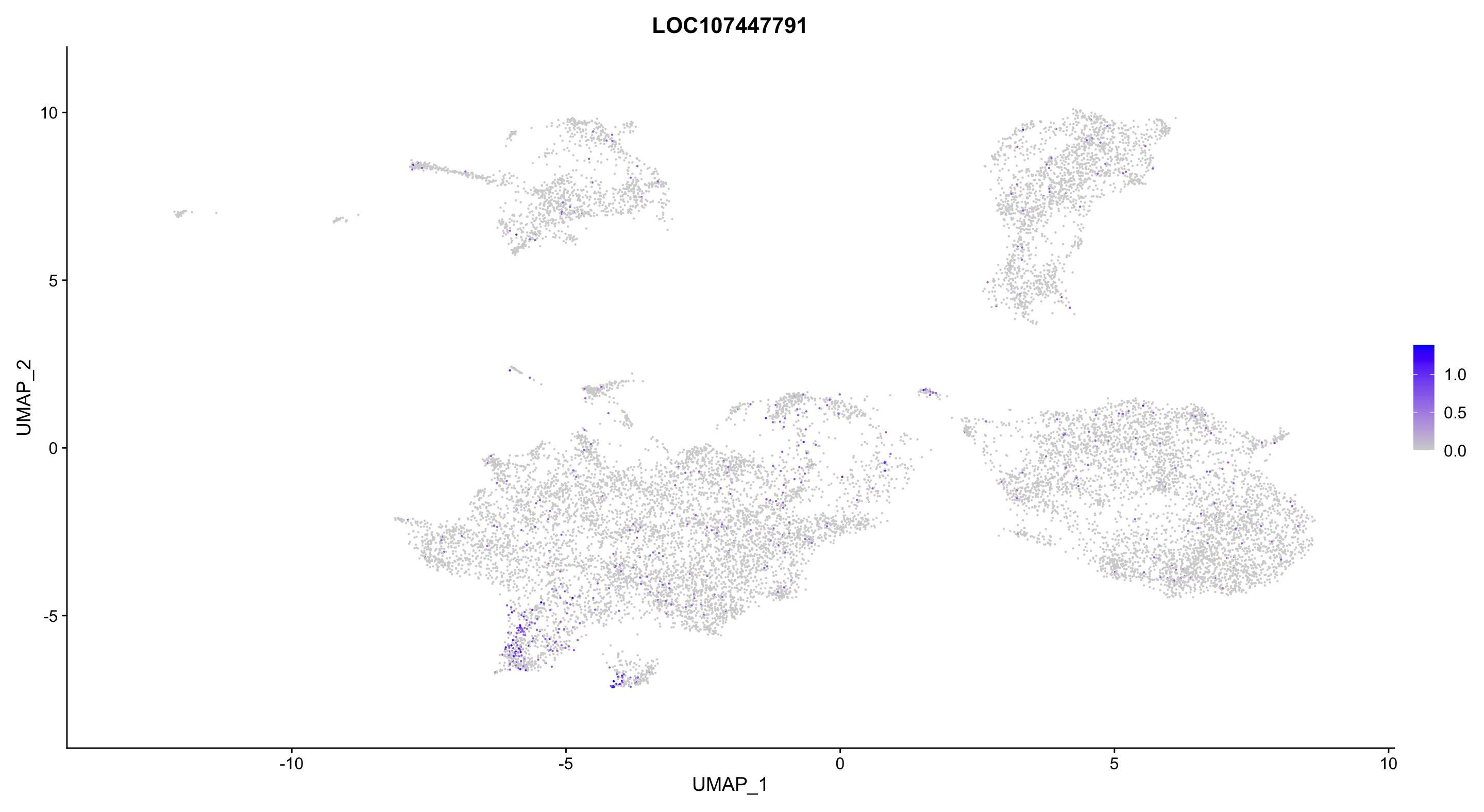

Supplement: Supplementary file 13 — Additional file 13: GO analysis results barplots (zipped folder) [file 13227_2024_230_MOESM13_ESM.zip › Supplementary File 14 - feature plots of C32 markers/23.Pt-unc7791 LOC107447791.png]

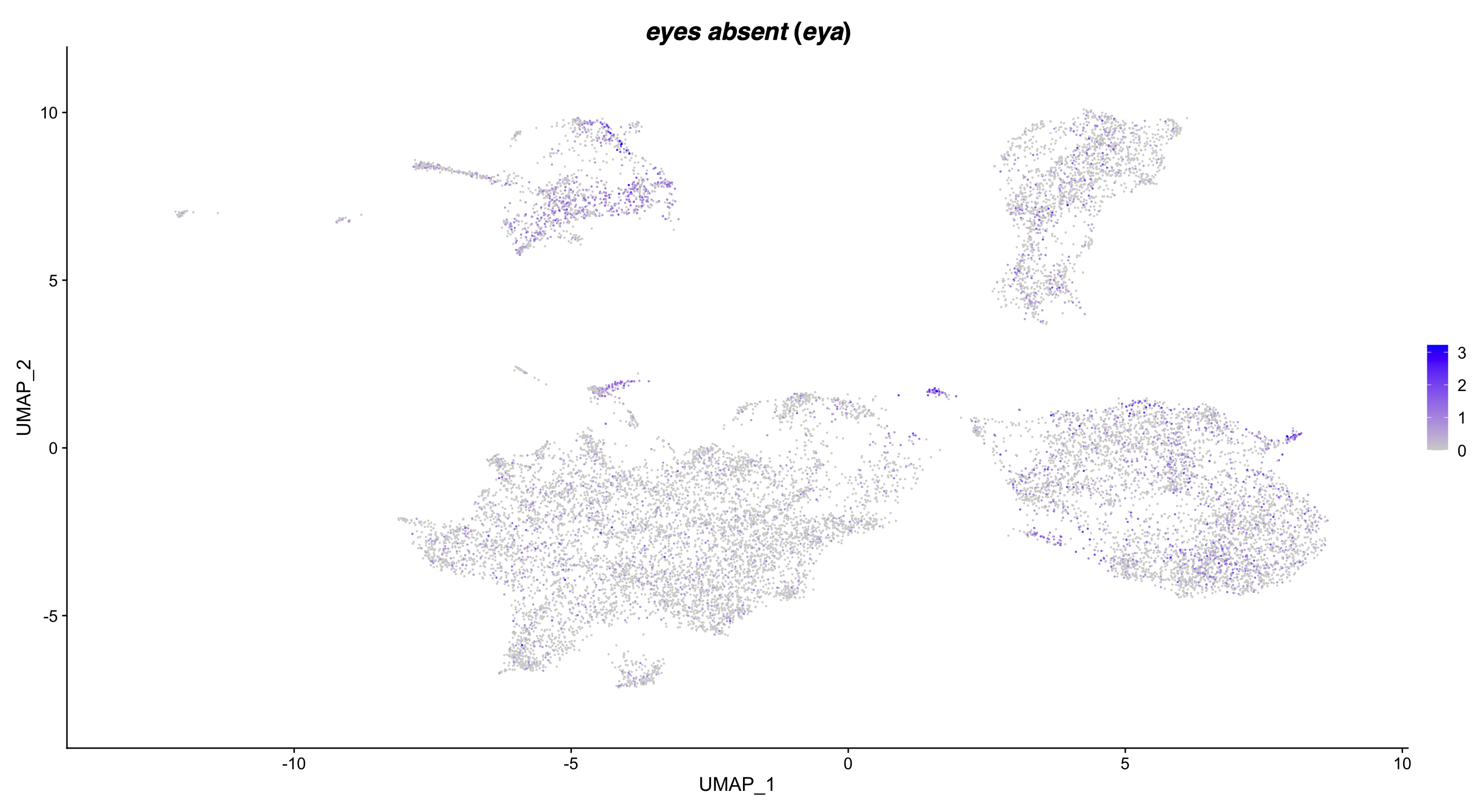

Supplement: Supplementary file 13 — Additional file 13: GO analysis results barplots (zipped folder) [file 13227_2024_230_MOESM13_ESM.zip › Supplementary File 14 - feature plots of C32 markers/6.Pt-eya LOC107452693.png]

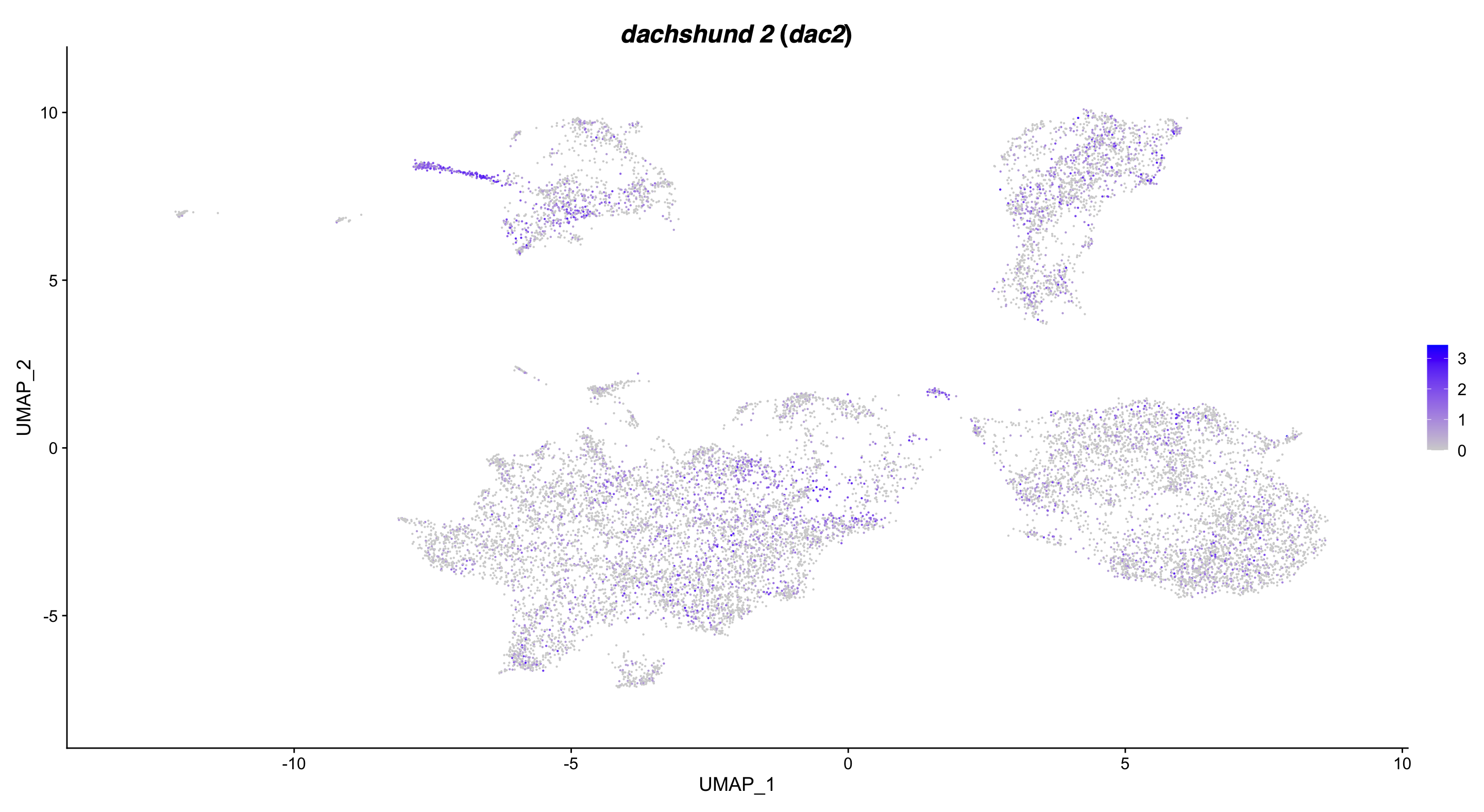

Supplement: Supplementary file 13 — Additional file 13: GO analysis results barplots (zipped folder) [file 13227_2024_230_MOESM13_ESM.zip › Supplementary File 14 - feature plots of C32 markers/15.Pt-dac2 LOC107452541.png]

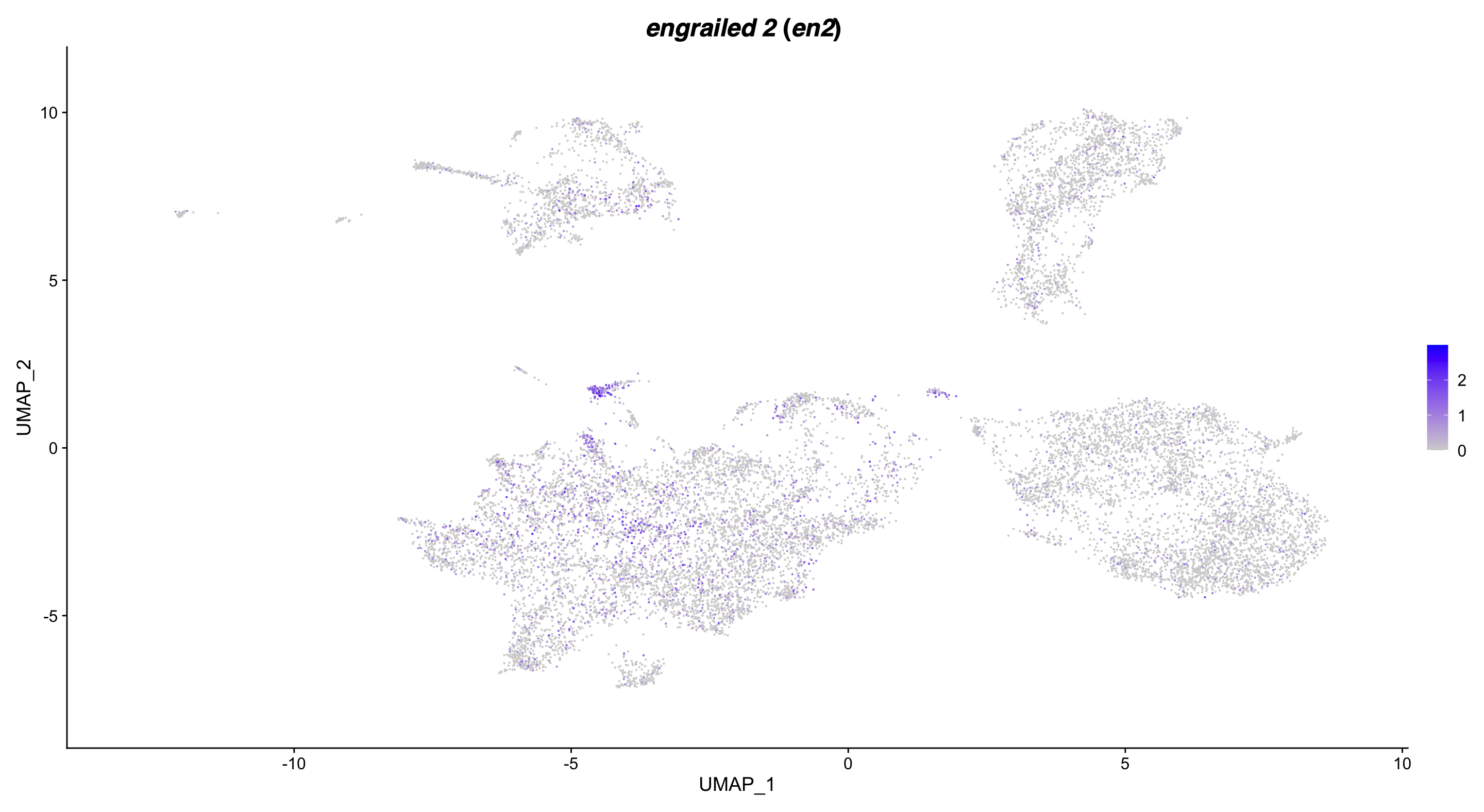

Supplement: Supplementary file 13 — Additional file 13: GO analysis results barplots (zipped folder) [file 13227_2024_230_MOESM13_ESM.zip › Supplementary File 14 - feature plots of C32 markers/12.Pt-en2 LOC107441561.png]

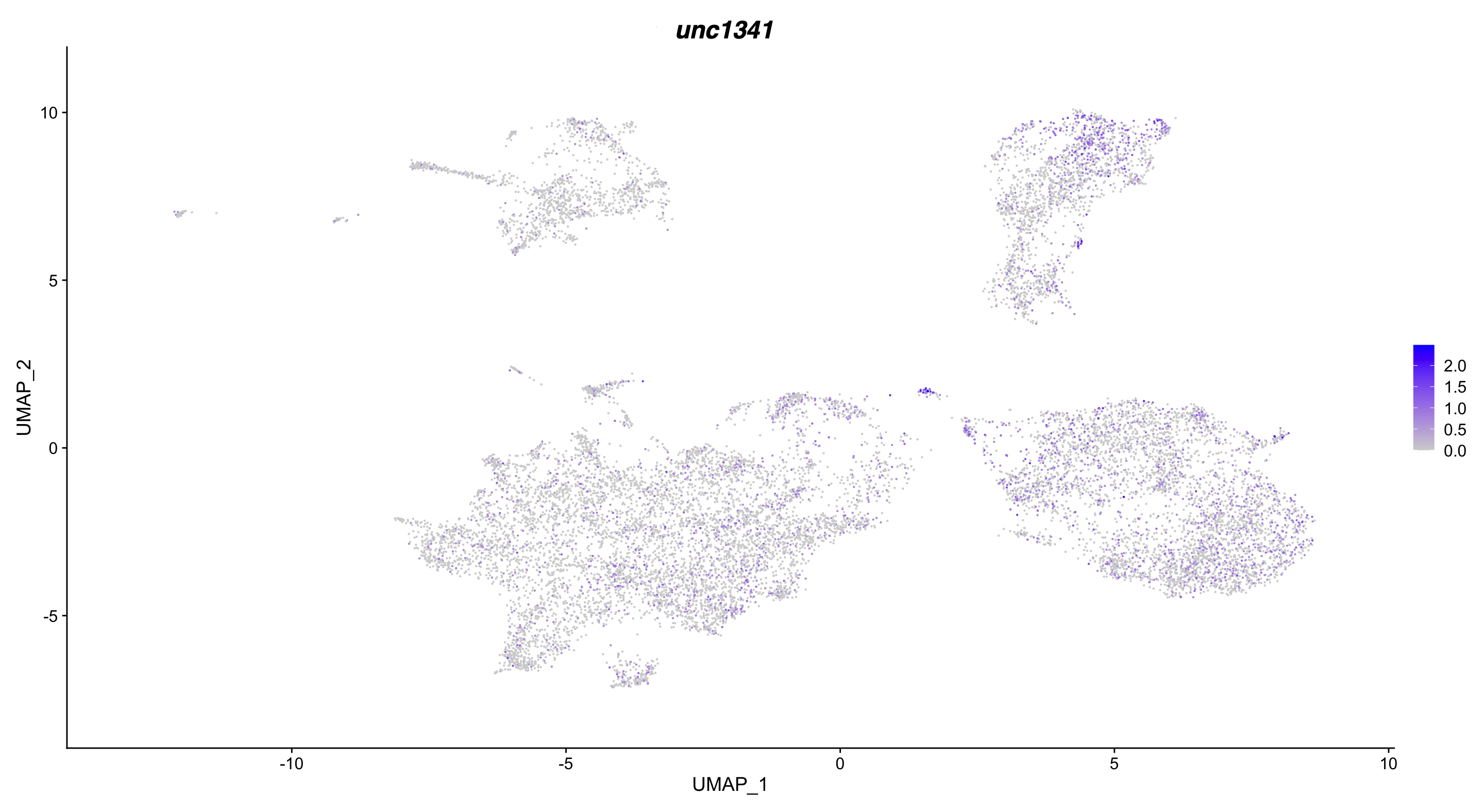

Supplement: Supplementary file 13 — Additional file 13: GO analysis results barplots (zipped folder) [file 13227_2024_230_MOESM13_ESM.zip › Supplementary File 14 - feature plots of C32 markers/18.Pt-unc1341 LOC122271341.png]

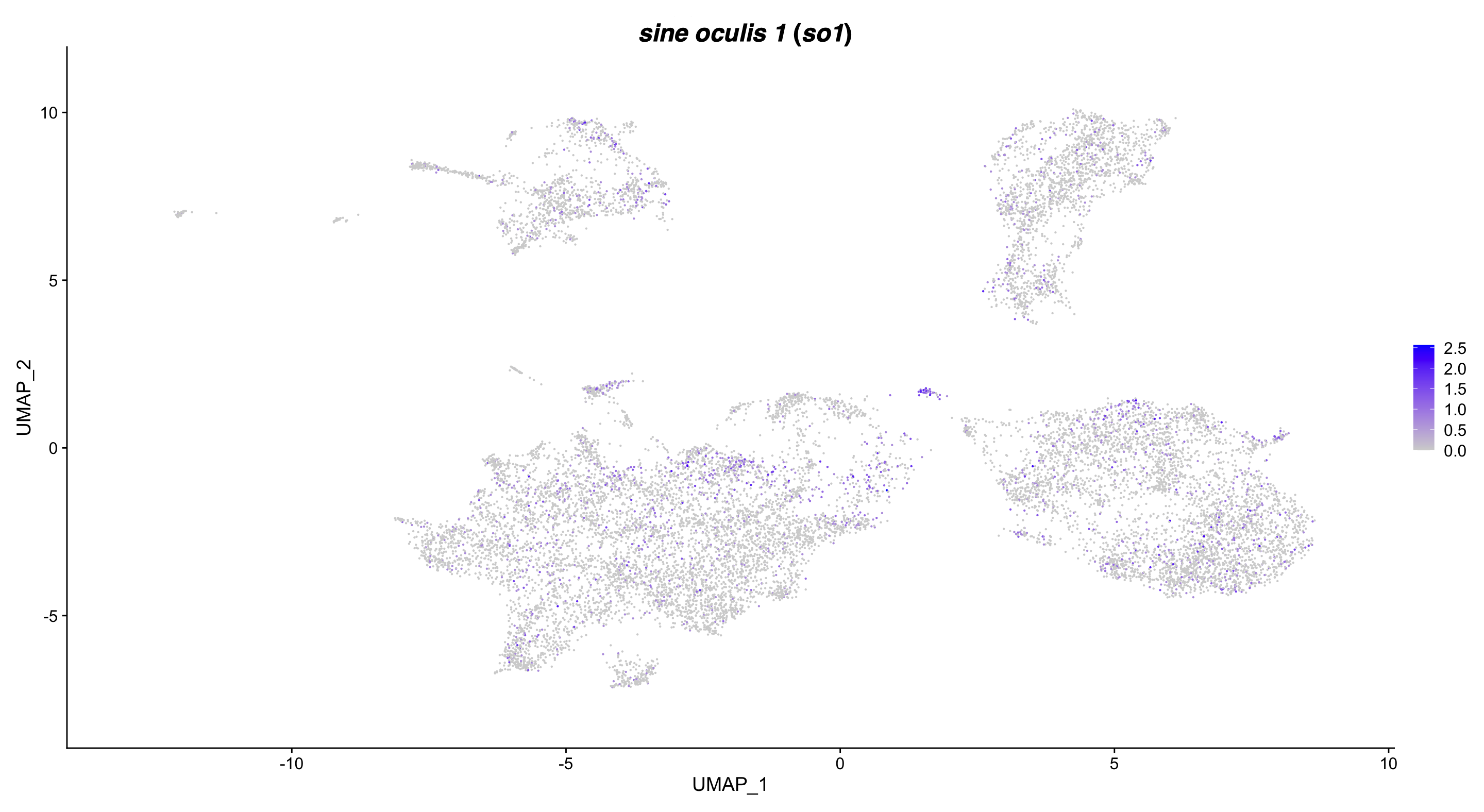

Supplement: Supplementary file 13 — Additional file 13: GO analysis results barplots (zipped folder) [file 13227_2024_230_MOESM13_ESM.zip › Supplementary File 14 - feature plots of C32 markers/5.Pt-so1 LOC107455071.png]

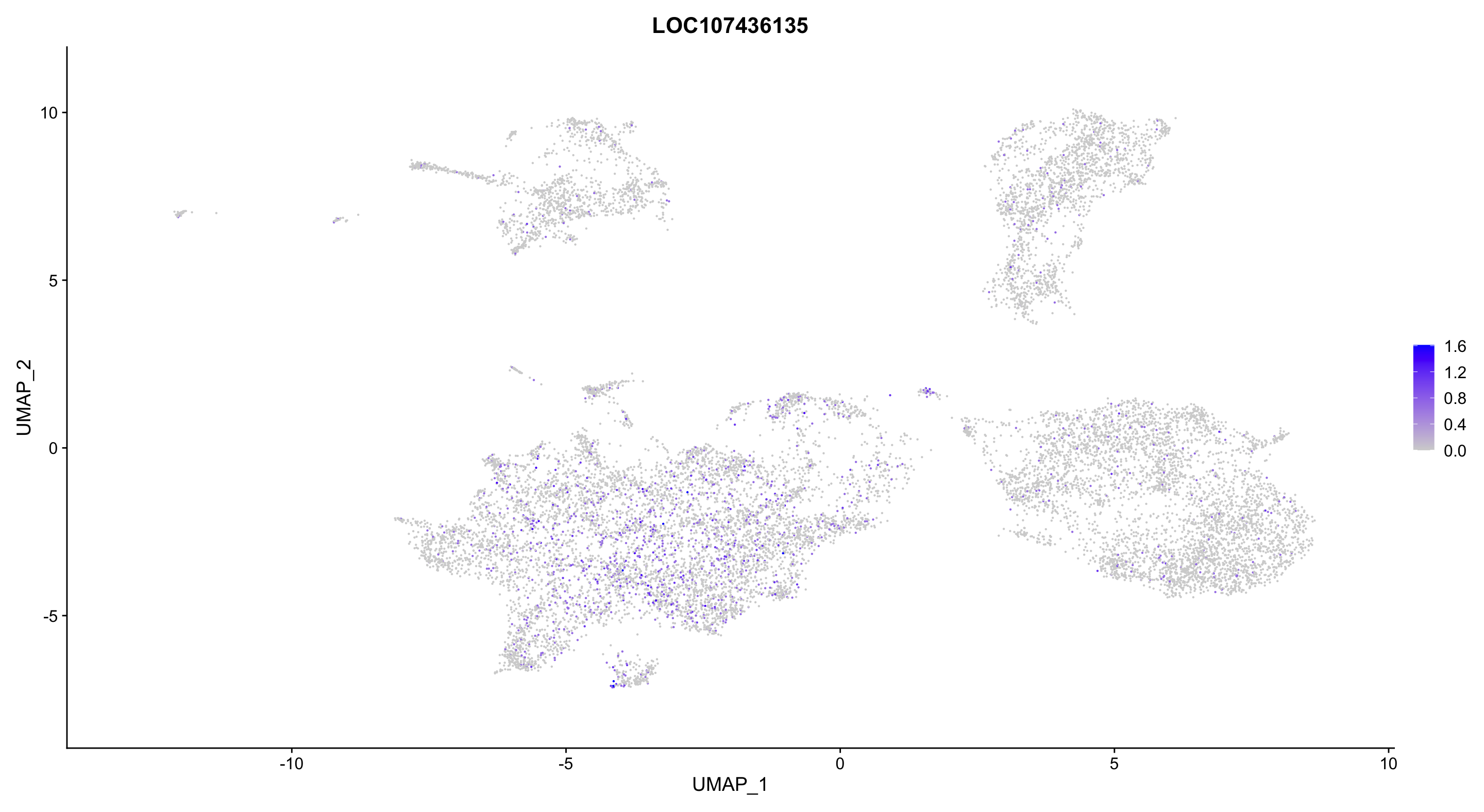

Supplement: Supplementary file 13 — Additional file 13: GO analysis results barplots (zipped folder) [file 13227_2024_230_MOESM13_ESM.zip › Supplementary File 14 - feature plots of C32 markers/22.Pt-NT1 LOC107436135.png]

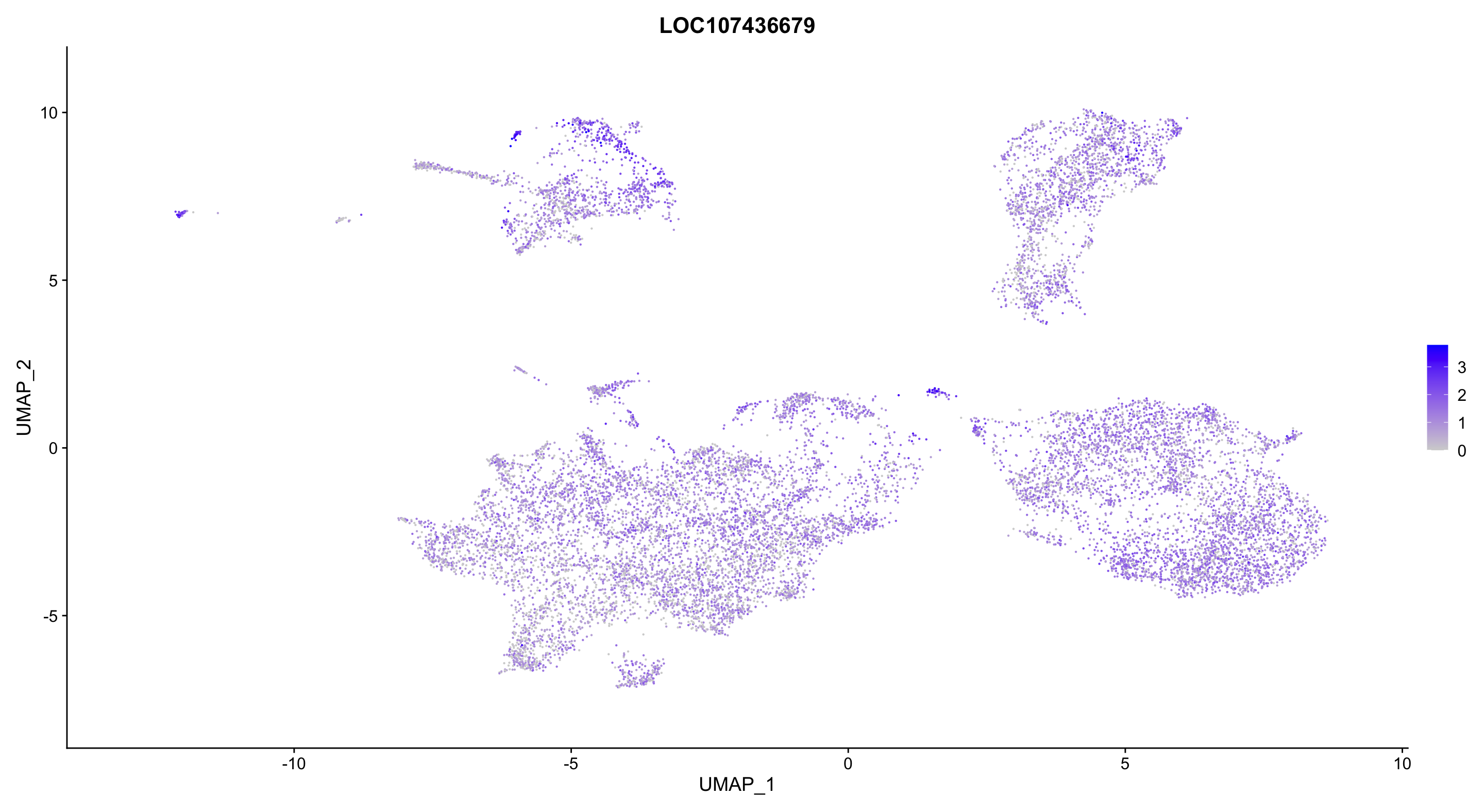

Supplement: Supplementary file 13 — Additional file 13: GO analysis results barplots (zipped folder) [file 13227_2024_230_MOESM13_ESM.zip › Supplementary File 14 - feature plots of C32 markers/14.Pt-Rbp6-like LOC107436679.png]

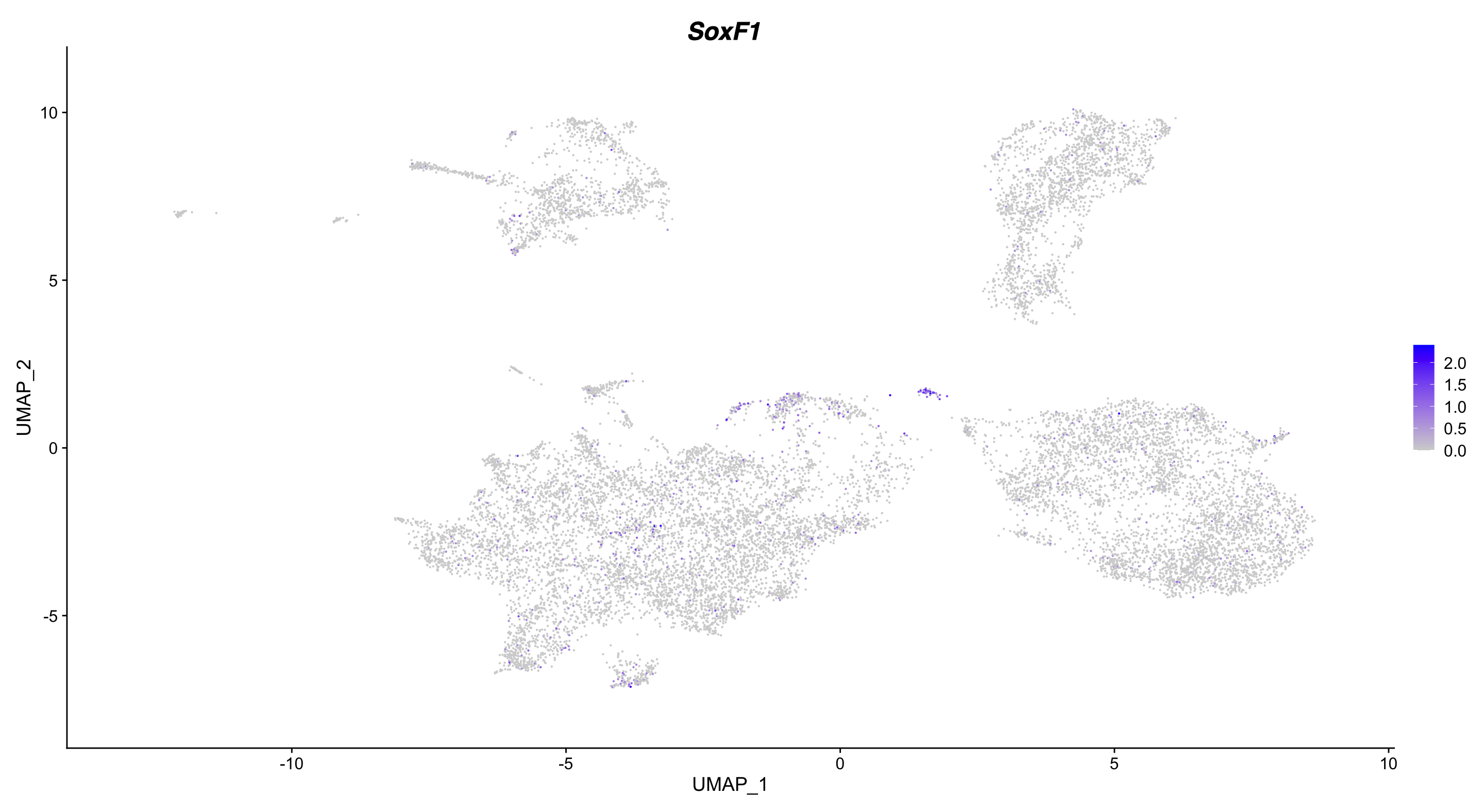

Supplement: Supplementary file 13 — Additional file 13: GO analysis results barplots (zipped folder) [file 13227_2024_230_MOESM13_ESM.zip › Supplementary File 14 - feature plots of C32 markers/2.Pt-SoxF1 LOC107446198.png]

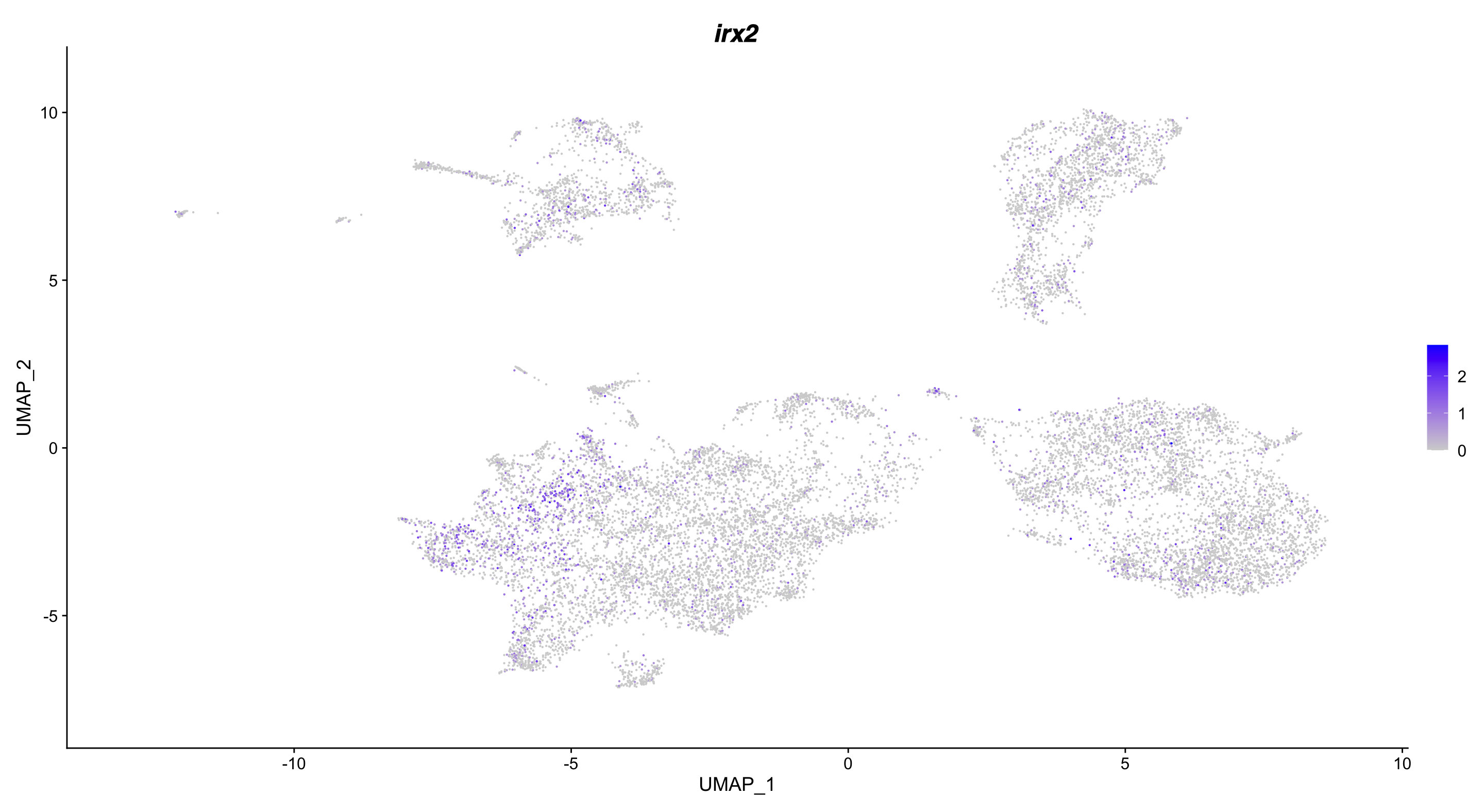

Supplement: Supplementary file 13 — Additional file 13: GO analysis results barplots (zipped folder) [file 13227_2024_230_MOESM13_ESM.zip › Supplementary File 14 - feature plots of C32 markers/24.Pt-irx2 LOC107439315.png]

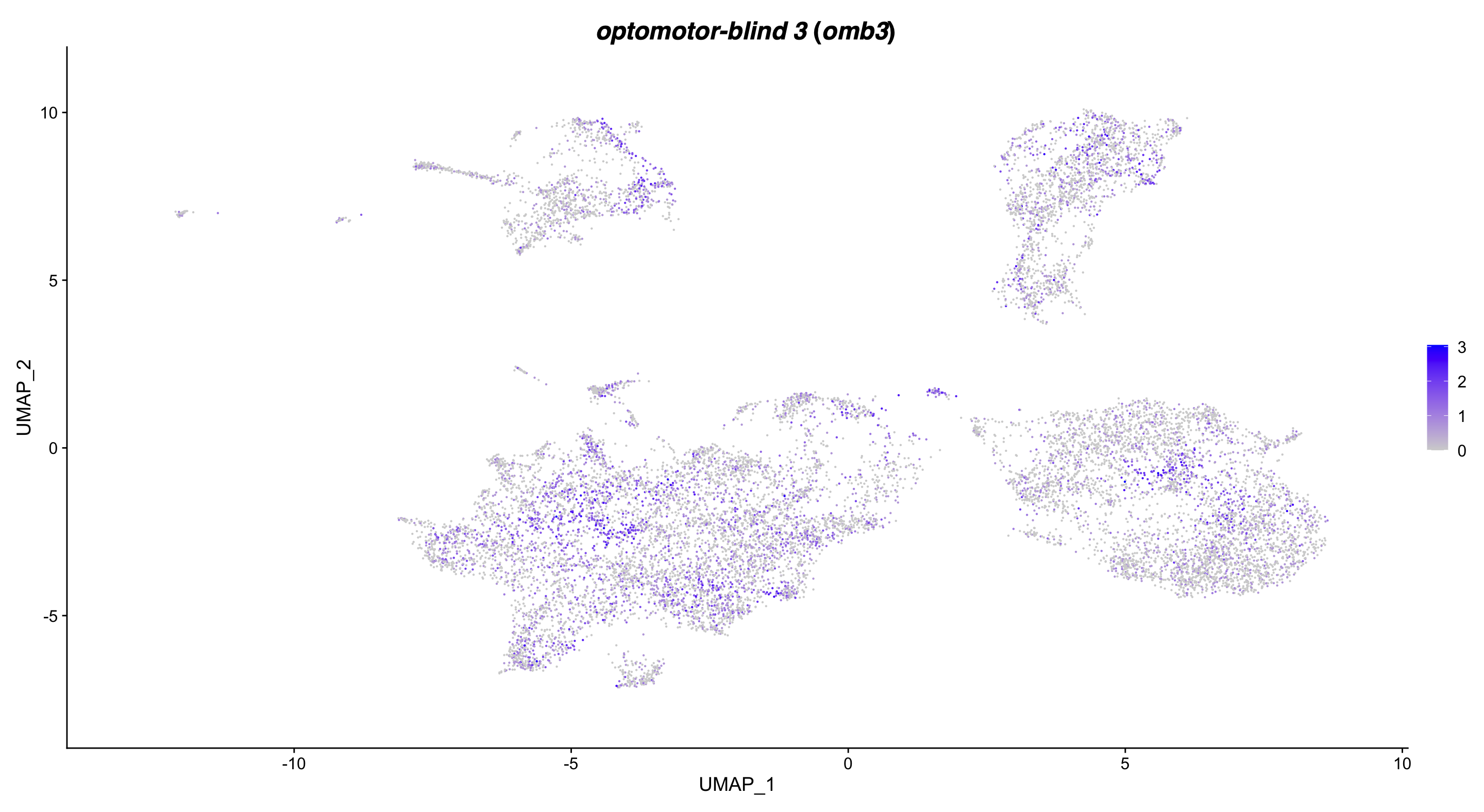

Supplement: Supplementary file 13 — Additional file 13: GO analysis results barplots (zipped folder) [file 13227_2024_230_MOESM13_ESM.zip › Supplementary File 14 - feature plots of C32 markers/17.Pt-omb3 LOC107451108.png]

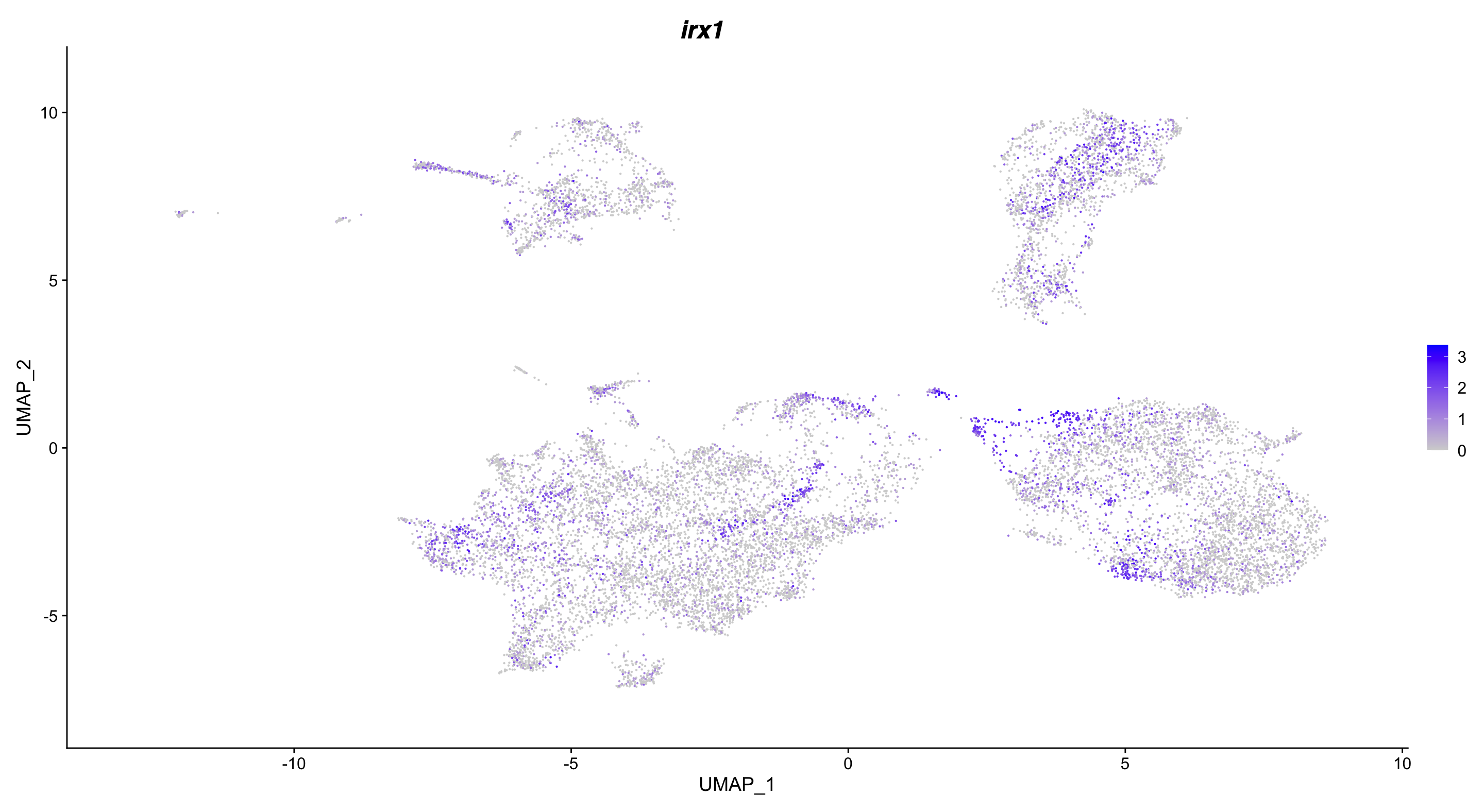

Supplement: Supplementary file 13 — Additional file 13: GO analysis results barplots (zipped folder) [file 13227_2024_230_MOESM13_ESM.zip › Supplementary File 14 - feature plots of C32 markers/7.Pt-irx1 LOC107437851.png]

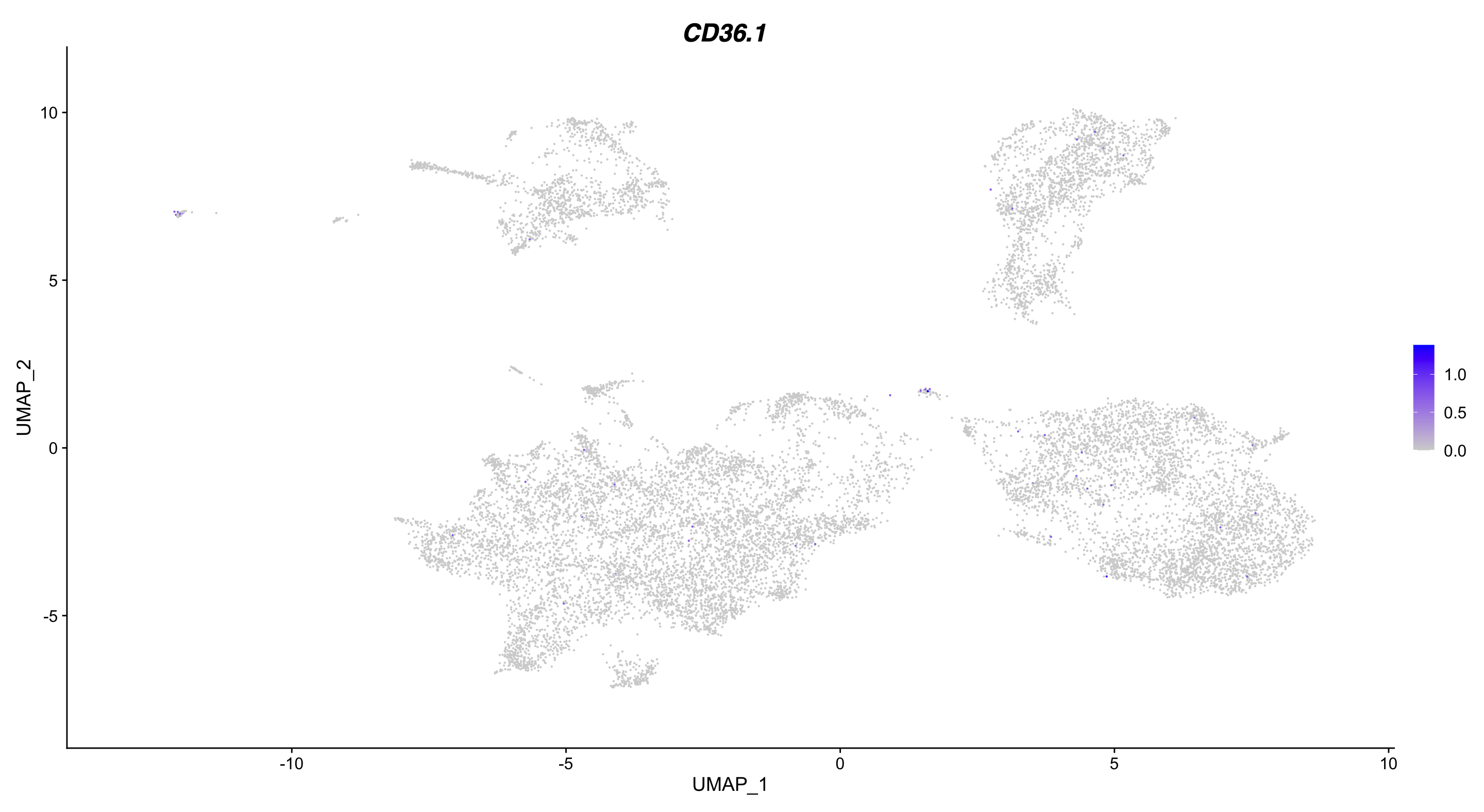

Supplement: Supplementary file 13 — Additional file 13: GO analysis results barplots (zipped folder) [file 13227_2024_230_MOESM13_ESM.zip › Supplementary File 14 - feature plots of C32 markers/4.Pt-CD36.1 LOC122271437.png]

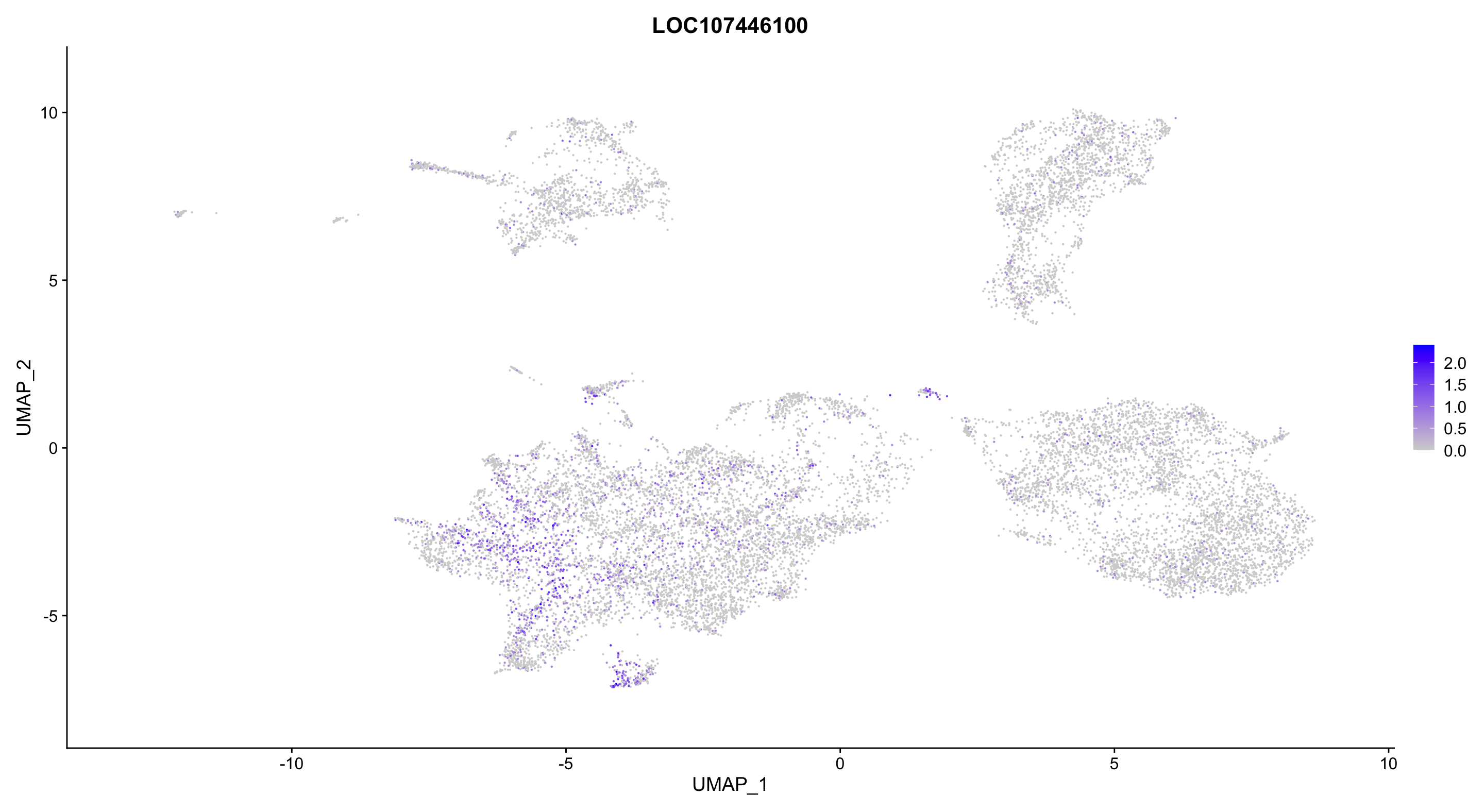

Supplement: Supplementary file 13 — Additional file 13: GO analysis results barplots (zipped folder) [file 13227_2024_230_MOESM13_ESM.zip › Supplementary File 14 - feature plots of C32 markers/8.Pt-mfas-like LOC107446100.png]

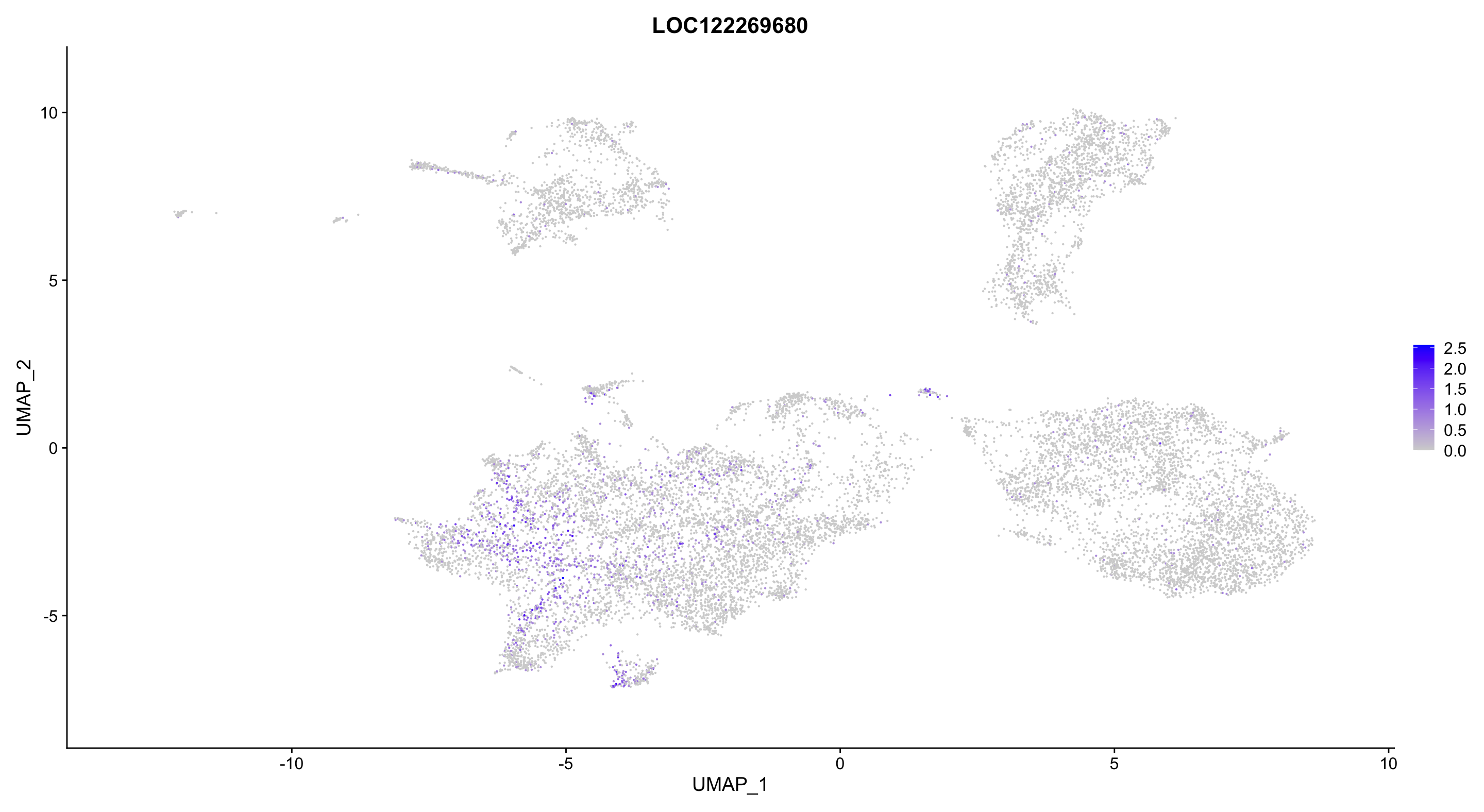

Supplement: Supplementary file 13 — Additional file 13: GO analysis results barplots (zipped folder) [file 13227_2024_230_MOESM13_ESM.zip › Supplementary File 14 - feature plots of C32 markers/11.Pt-unc9680 LOC122269680.png]

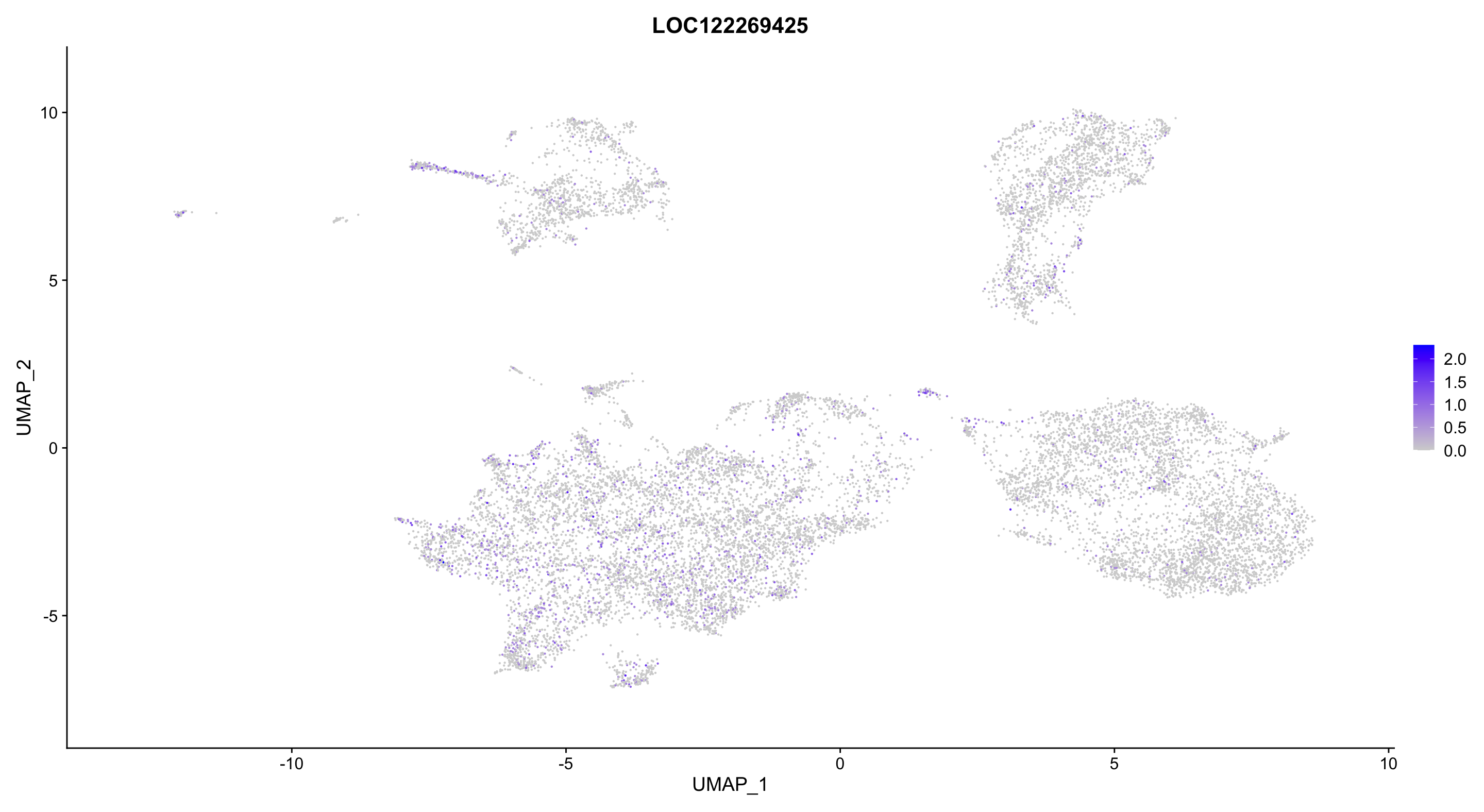

Supplement: Supplementary file 13 — Additional file 13: GO analysis results barplots (zipped folder) [file 13227_2024_230_MOESM13_ESM.zip › Supplementary File 14 - feature plots of C32 markers/20.Pt-unc9425 LOC122269425.png]

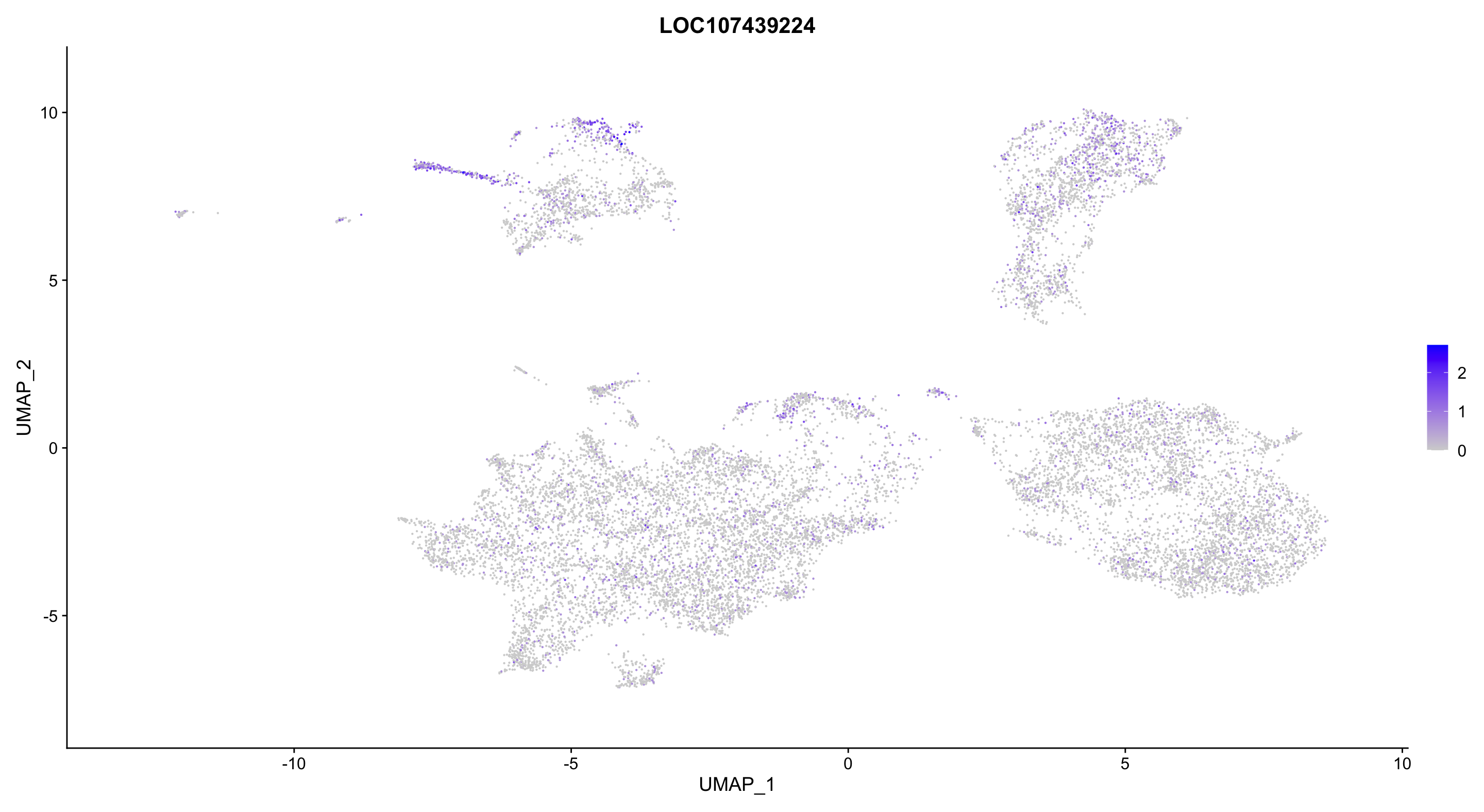

Supplement: Supplementary file 13 — Additional file 13: GO analysis results barplots (zipped folder) [file 13227_2024_230_MOESM13_ESM.zip › Supplementary File 14 - feature plots of C32 markers/16.Pt-SKIP-like LOC107439224.png]

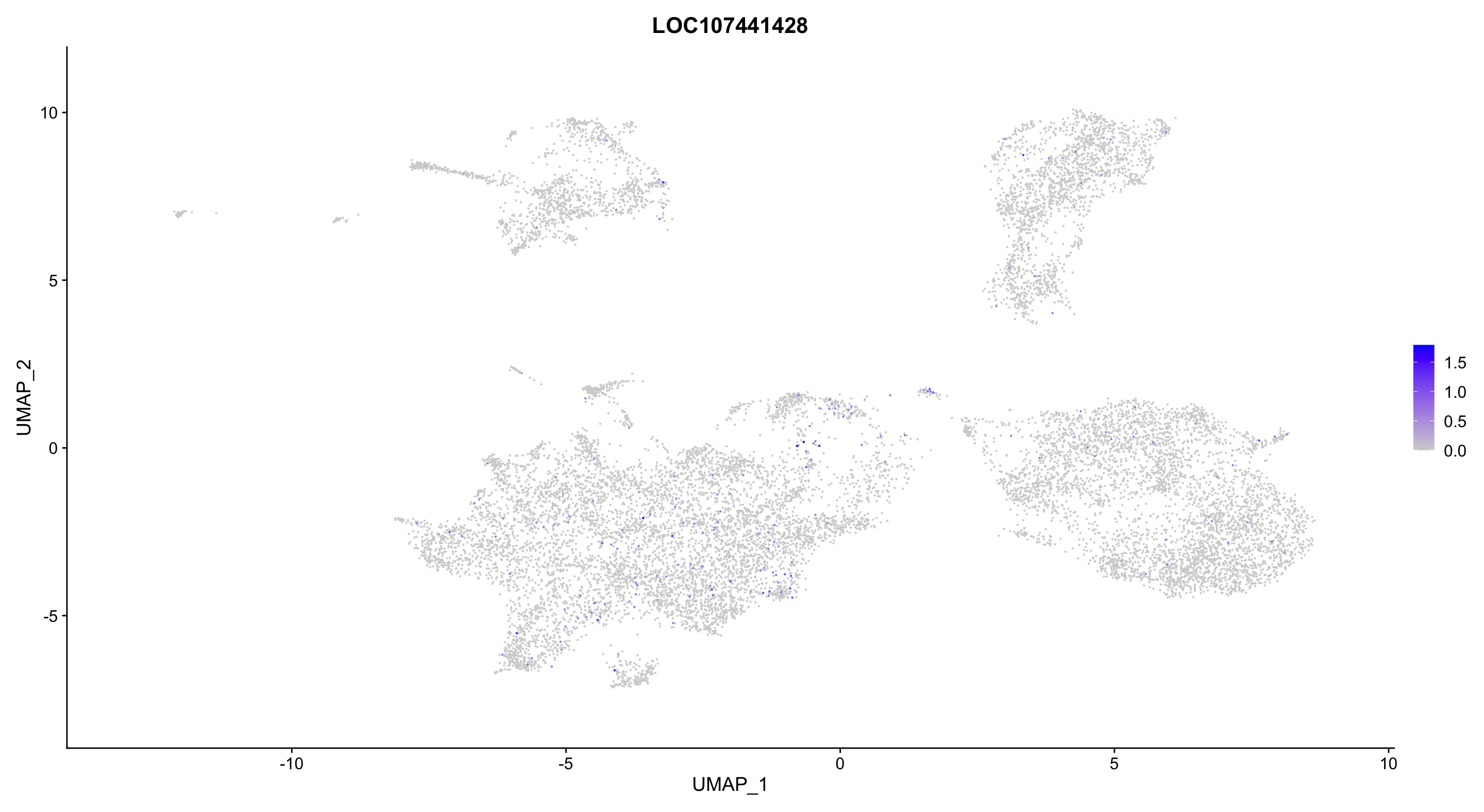

Supplement: Supplementary file 13 — Additional file 13: GO analysis results barplots (zipped folder) [file 13227_2024_230_MOESM13_ESM.zip › Supplementary File 14 - feature plots of C32 markers/9.Pt-CG13875-like LOC107441428.png]

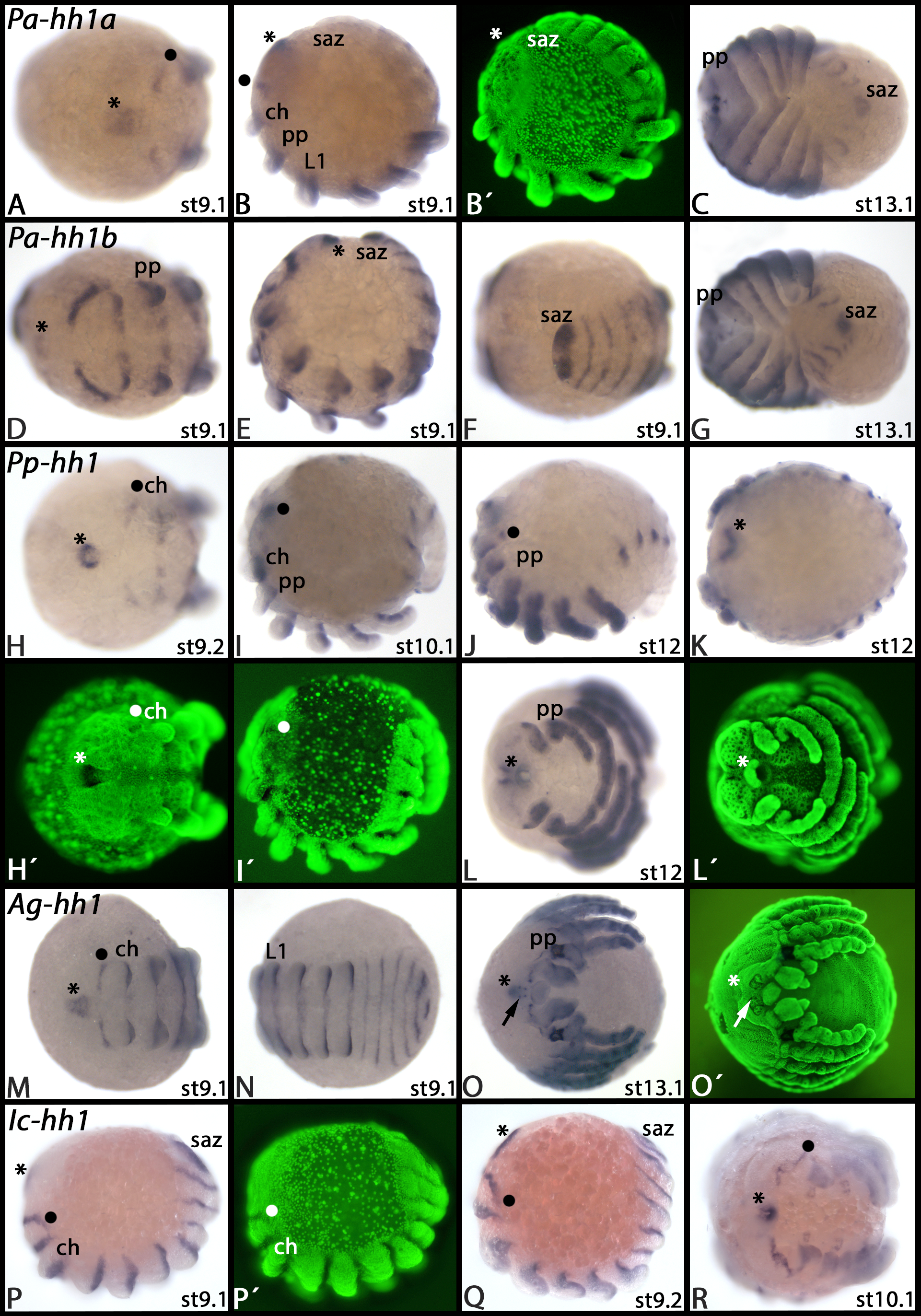

Supplement: Supplementary file 14 — Additional file 14: Feature plots of all markers of cluster C32 (zipped folder) [file 13227_2024_230_MOESM14_ESM.tif]

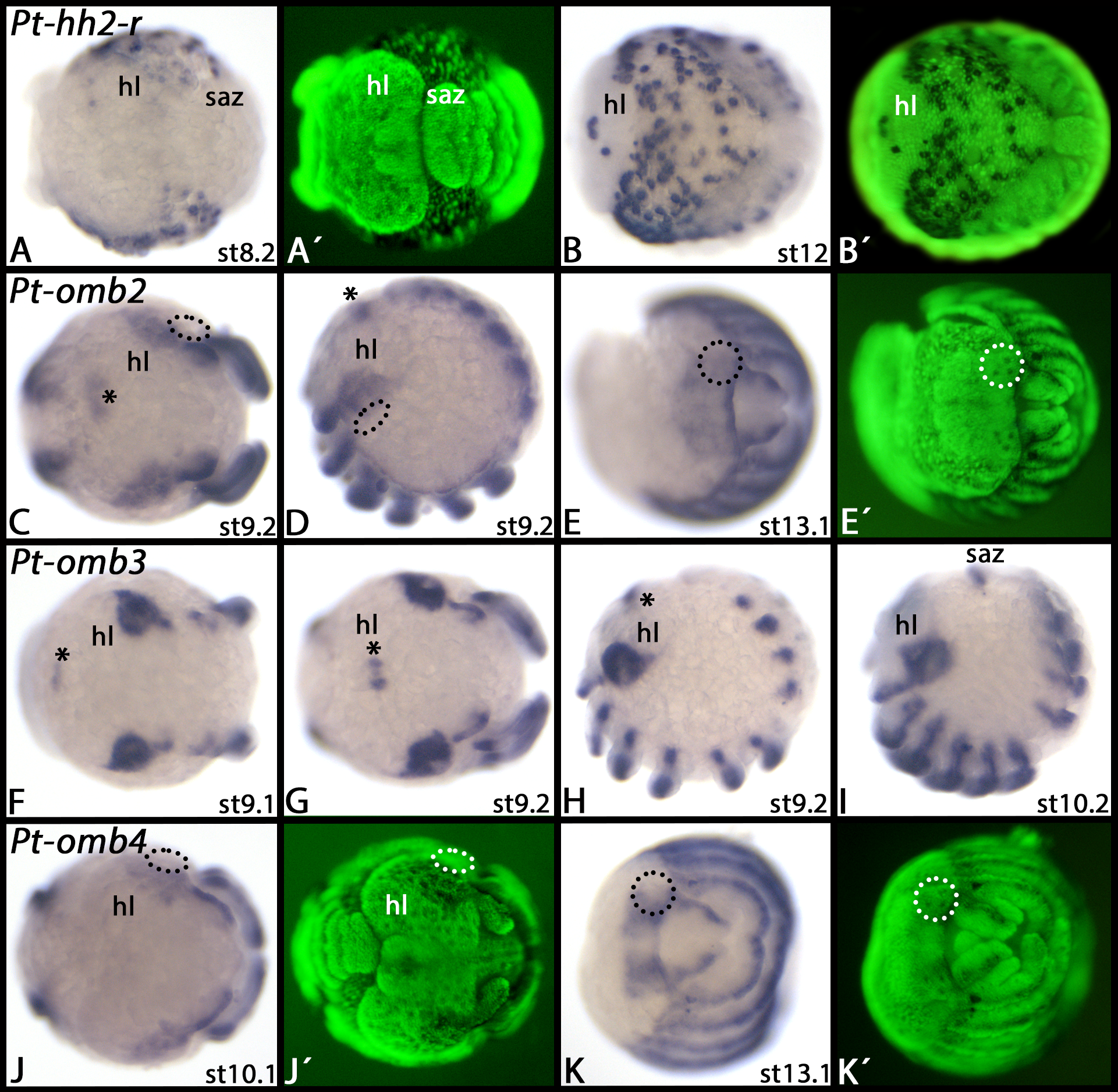

Supplement: Supplementary file 15 — Additional file 15: Expression of spider hh1 genes. In all panels, anterior is to the left. Panels A, C, D, F, G, H, L, M, N, O and R represent lateral views. Panels B, E, I, J, P and Q represent lateral views (in these panels, dorsal is up). Panel K represents a dorsal view. Note the segment-polarity gene-like expression of all hh1-group genes, expression along the appendages, expression in the segment addition zone (saz), and expression in the anlagen of the stomodaeum (asterisks in all panels). The arrow in panel O points to four dots of expression in the neuronal ectoderm that thus are not associated with the developing eyes. In all panels, full circles mark expression in the pre-cheliceral region, but note that this expression is not at the place where the eyes will form. Panels B´, H´, I´, L´, O´ and P´ represent Sybr-green staining of corresponding embryos. Developmental stages are indicated after Mittmann and Wolff [61]; note that developmental stages in different species of spiders have been defined by comparable morphological landmarks such as the overall shape of the embryo and the length of the appendages. Abbreviations: ch, cheliceral segment; L, leg-bearing segment; pp, pedipalp-bearing segment; saz, segment addition zone. [file 13227_2024_230_MOESM15_ESM.tif]
