# Supplementary material for: Single-cell sequencing suggests a conserved function of Hedgehog-signalling in spider eye development
Source: EvoDevo. 2024 Sep 26;15:11. doi: 10.1186/s13227-024-00230-6 (PMC11428483; doi:10.1186/s13227-024-00230-6)
Supplement: Supplementary file 12 — Additional file 12: Info_number_orthologs_per_cluster (WORD file: Table) [file 13227_2024_230_MOESM12_ESM.docx]

| Cluster | Number of markers | Protein-coding  genes | *Drosophila* orthologs (RBH method) | GO: Biological Process terms |
| --- | --- | --- | --- | --- |
| C0 | 310 | 303 | 156 | 144 |
| C1 | 51 | 47 | 32 | 31 |
| C2 | 65 | 62 | 33 | 32 |
| C3 | 20 | 20 | 13 | 13 |
| C4 | 54 | 48 | 30 | 28 |
| C5 | 56 | 53 | 29 | 26 |
| C6 | 75 | 74 | 39 | 37 |
| C7 | 303 | 299 | 158 | 139 |
| C8 | 151 | 148 | 69 | 61 |
| C9 | 9 | 5 | 3 | 3 |
| C10 | 26 | 25 | 16 | 16 |
| C11 | 137 | 132 | 75 | 67 |
| C12 | 125 | 118 | 60 | 59 |
| C13 | 168 | 163 | 61 | 52 |
| C14 | 37 | 35 | 17 | 17 |
| C15 | 81 | 74 | 45 | 44 |
| C16 | 191 | 177 | 102 | 97 |
| C17 | 84 | 77 | 38 | 34 |
| C18 | 187 | 184 | 101 | 96 |
| C19 | 25 | 22 | 13 | 13 |
| C20 | 119 | 118 | 61 | 55 |
| C21 | 159 | 152 | 67 | 60 |
| C22 | 71 | 71 | 32 | 29 |
| C23 | 38 | 37 | 21 | 21 |
| C24 | 14 | 14 | 9 | 9 |
| C25 | 20 | 20 | 11 | 11 |
| C26 | 39 | 36 | 16 | 15 |
| C27 | 25 | 24 | 13 | 13 |
| C28 | 81 | 77 | 38 | 30 |
| C29 | 11 | 8 | 3 | 2 |
| C30 | 234 | 227 | 94 | 84 |
| C31 | 96 | 91 | 41 | 37 |
| C32 | 25 | 23 | 11 | 10 |
| C33 | 81 | 78 | 23 | 22 |
| C34 | 123 | 115 | 30 | 25 |
